# Nanoparticles that modulate immune cells - an important strategy for the future treatment of tumors

**DOI:** 10.3389/fimmu.2026.1803646

**Published:** 2026-04-22

**Authors:** Yan Zhao, Guoxing Zhang, Xin Nie, Jingyuan Ren, Dequan Xu, Hongbo Yan, Jianxin Zhao, Laga Tong, Lei Zhao, Haifeng Liu

**Affiliations:** 1 Special Care Ward, Jilin Province Cancer Hospital, Changchun, Jilin, China; 2 Stroke center, Jilin Electric Power Hospital, Changchun, Jilin, China; 3 Department of Head and Neck Surgery, Jilin Province Cancer Hospital, Changchun, Jilin, China; 4 Department of Radiation Oncology, Jilin Province Cancer Hospital, Changchun, Jilin, China; 5 Department of breast Surgery, Jilin Province Cancer Hospital, Changchun, Jilin, China; 6 Center for Disease Control and Prevention, Horqin Right Middle Banner, Xing’an League, Inner Mongolia, China; 7 Department of Neurosurgery, Mongolian Medicine Hospital, Horqin Right Middle Banner, Xing’an League, Inner Mongolia, China; 8 The Second Middle School, Horqin Right Middle Banner, Xing’an League, Inner Mongolia, China

**Keywords:** immune cells, nanoparticles, targeted, tumor, tumor microenvironment

## Abstract

Cancer has become a major public health issue that seriously threatens human health. With the widespread development and application of drug-loaded nanosystems in tumor therapy, numerous studies have confirmed that various nanoparticles can exert anti-tumor activity by regulating one or more immune cells in the tumor microenvironment (TME), including depleting M2-type TAMs in the TME, limiting the recruitment and localization of TAMs, reprogramming M2-type TAMs into M1-type TAMs, enhancing NK cells homing or function, promoting DCs maturation, inhibiting TANs recruitment or altering their polarity, reducing circulating and tumor-infiltrating MDSCs, altering MDSCs phenotype or inhibiting MDSCs functions (including nanocapsules, metal-organic frameworks, micelles, polymers, dendritic macromolecules, liposomes, and other material nanoparticles), promoting CD8^+^ T cells proliferation or activity and reducing the number of Tregs (including nanoparticles composed of liposomes, gels, cerium, and selenium, hyaluronic acid-modified nanoparticles, nanocapsules, nanovesicles, other material nanoparticles, and nanoparticles combined with other treatment modalities), besides, some nanoparticles can exert anti-tumor activity by regulating two or more types of immune cells in the TME. In short, nanoparticles targeting immune cells will become an important strategy in tumor treatment based on the advantages of nanoparticles in enhancing efficacy and reducing toxicity in tumor therapy, as well as the important role of immune cells in tumor occurrence and development. In this article, we will provide a detailed introduction to nanoparticles that exert anti-tumor activity by regulating immune cells in the TME and briefly introduce the mechanisms by which nanoparticles regulate immune cells.

## 1 Introduction

Solid tumors are made up of tumor cells and the tumor microenvironment (TME) that includes various cells (immune cells and mesenchymal cells) and extracellular matrix (ECM) (1, 2). And people have gradually realized the importance of immune cells in TME in anti-tumor therapy with the discovery of immune checkpoints and their significant efficacy in anti-tumor therapy (3), the immune cells in TME mainly include tumor-associated macrophages (TAMs), natural killer (NK) cells, dendritic cells (DCs), tumor-associated neutrophils (TANs), myeloid-derived suppressor cells (MDSCs) and T cells (4). The immune cells in TME are mainly immunosuppressive cells with the progression of tumors, such as M2 TAMs, MDSCs, N2 TANs, and regulatory T cells (Tregs), while the number of anti-tumor immune cells decreases and their functions are suppressed, such as DCs, NK cells, M1 TAMs, and N1 TANs, which leads to tumor progression and recurrence due to tumor cells evading the surveillance of the immune system (5). Based on this, targeted immune cell therapy for tumors can be achieved by increasing anti-tumor immunes cells or their function and reducing immunosuppressive cells or weakening their function. At present, T cell-targeting immune checkpoint inhibitors have been recommended by guidelines for significant efficacy, such as programmed cell death 1/programmed death-ligand1 (PD-1/PD-L1) antibodies, cytotoxic T lymphocyte–associated protein-4 (CTLA-4) antibodies, and chimeric antigen receptor T (CAR-T)in adoptive cell therapy (ACT). In addition, the strategy of targeting immune cells to treat tumors can also be achieved by reducing M2 TAMs, N2 TANs, MDSCs, and Tregs in the TME and reprogramming TAMs and TANs into tumor killer cells (5).

**Figure 1:**
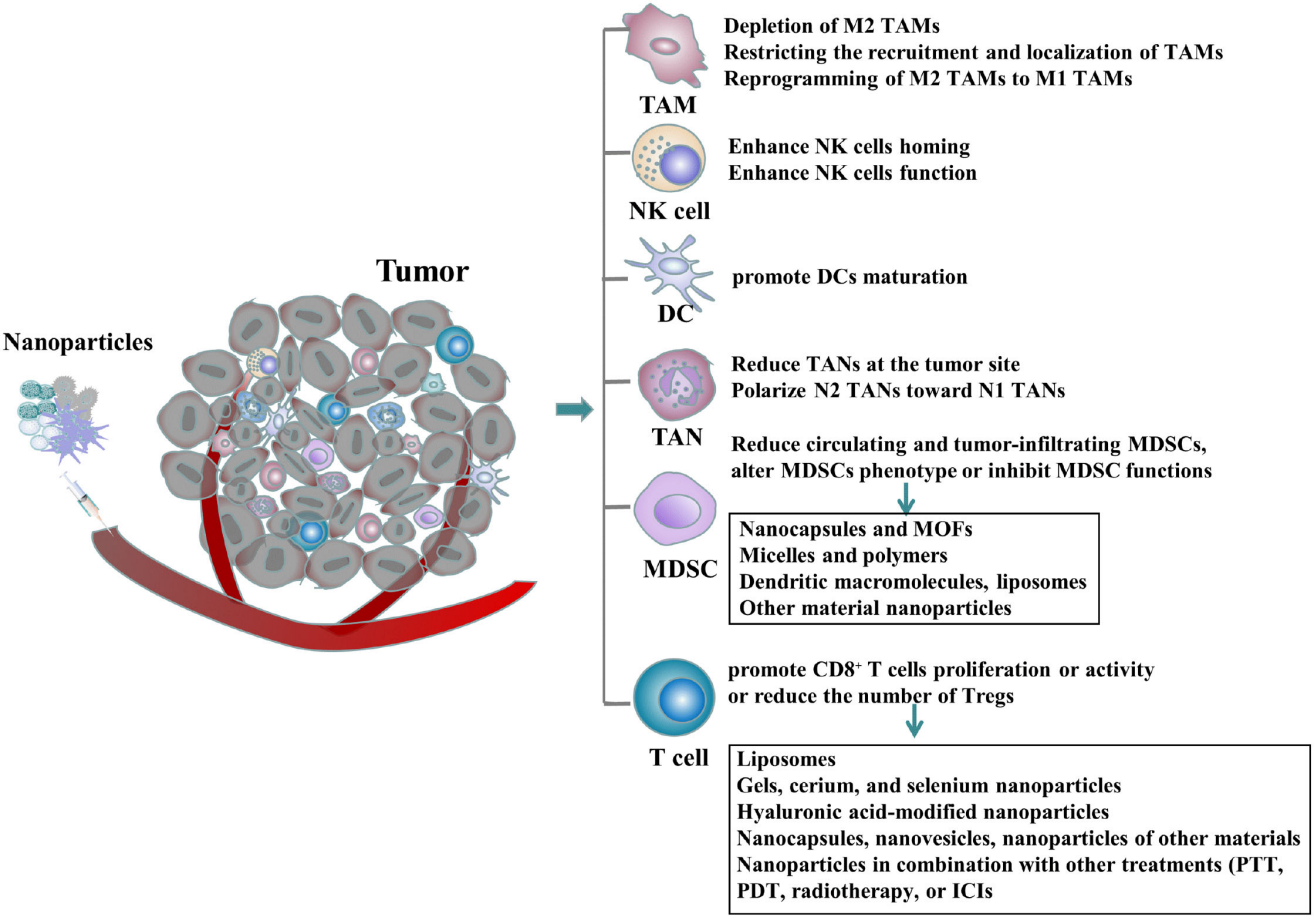
Nanoparticles can exert anti-tumor effects by modulating one or more immune cells in the tumor microenvironment:including depleting M2 TAMs in the TME, restricting the recruitment and localization of TAMs, reprogramming M2 TAMs into M1 TAMs, enhancing the homing or function of NK cells, promoting DCs maturation, inhibiting or reducing TANs at tumor sites or changing their polarization, reducing circulating and tumor-infiltrating MDSCs, altering MDSCs phenotype or inhibiting MDSCs function (including nanocapsules, metal-organic frameworks, micelles, polymers, dendrimers, liposomes, and nanoparticles of other materials), promoting the proliferation or activity of CD8^+^ T cells, and reducing the number of Tregs (including nanoparticles composed of liposomes, gels, cerium, and selenium, hyaluronic acid-modified nanoparticles, nanocapsules, nanovesicles, other material nanoparticles, and nanoparticles combined with other treatment modalities).

With the development of nanotechnology and the wide application of nano-loaded materials in the treatment of diseases, the nano-drug co-delivery system has shown strong advantages in the delivery of drugs, natural active ingredients and genes, such as improving the water solubility of loaded drugs, prolonging the drug cycle time, controlling the slow release of drugs and targeting tumors, and based on these properties of drug-loaded nanosystems make them show a wide range of applications in the treatment of tumors (6). Up to now, a variety of anti-tumor nanodrugs have been developed and some of them have been approved for clinical application and have shown strong anti-tumor activity and safety, such as liposomal doxorubicin, albumin-paclitaxel, ilinotecan liposome, etc (7–9). In addition, many nanoparticles with anti-tumor activity have been successfully synthesized and some nanoparticles have been proven to exert anti-tumor effects by regulating immune cells in TME, such as TAMs, NK cells, DCs, TANs, MDSCs, and T cells (**Figure 1**). Not only that, some nanoparticles can also exert anti-tumor activity by regulating two or more immune cells in the TME and we will introduce in detail the nanoparticles that exert anti-tumor activity by regulating immune cells in TME in this article (**Table 1**).

## 2 Nanoparticles regulate TAMs

**Figure 2:**
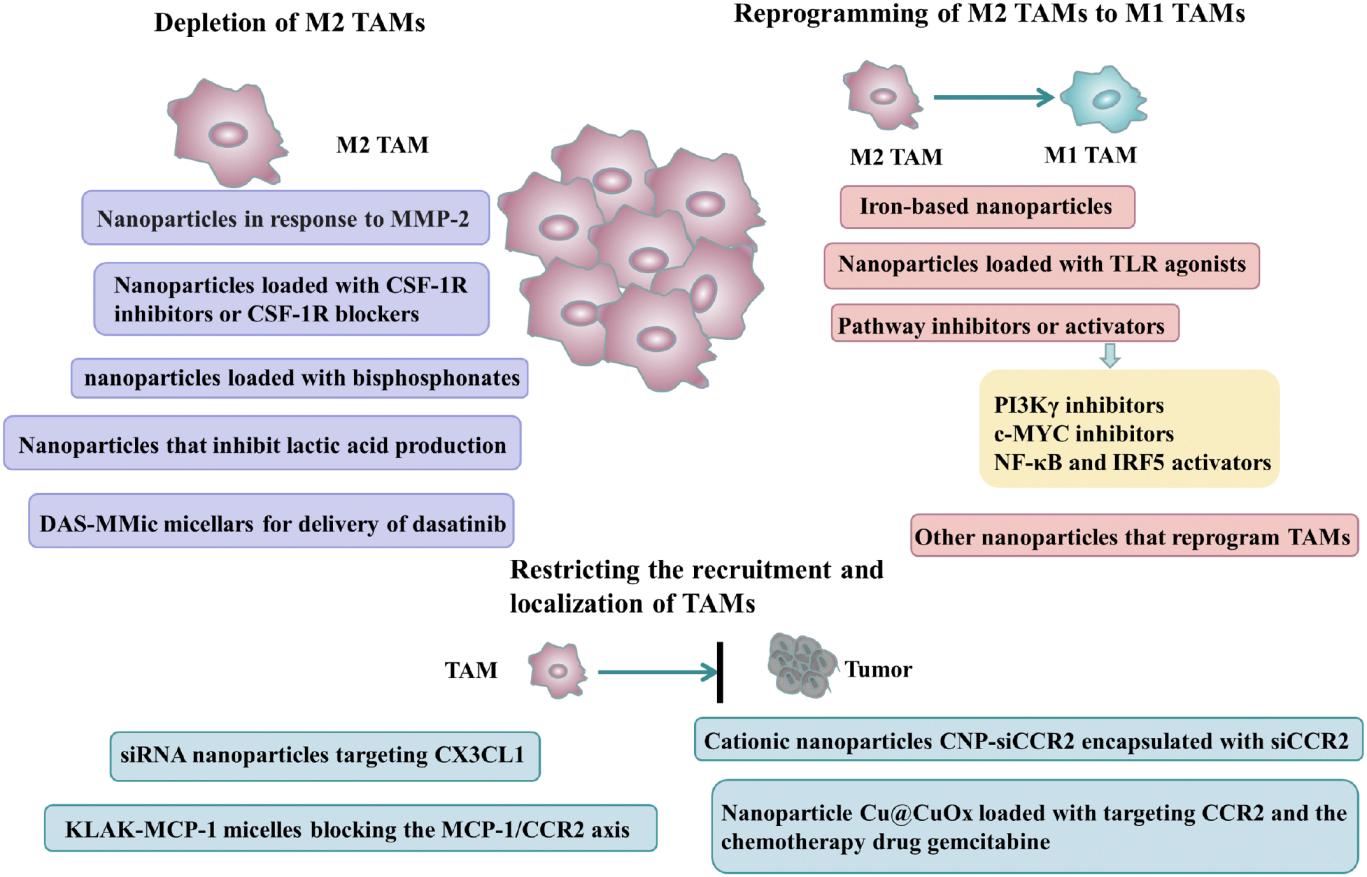
Nanoparticles targeting TAMs to exert anti-tumor effects: including nanoparticles that deplete M2-type TAMs, nanoparticles that restrict the recruitment and localization of TAMs, and nanoparticles that reprogram M2-type TAMs into M1-type TAMs.

TAMs are the most abundant immune cells in TME and the macrophages in TME are divided into two subtypes, namely M1 TAMs with tumor inhibition and antitumor angiogenesis and M2 TAMs with tumor promogenesis, metastasis and tumor angiogenesis, and based on this TAMs can be used as a potential target for cancer therapy (10, 11). With the application of nanomaterials in anti-tumor therapy, a variety of nanoparticles have been shown to exert anti-tumor effects by targeting TAMs, including but not limited to limit the survival of TAMs, inhibit TAMs recruitment to tumors, and “reprogram” TAMs from tumor-promoting M2 TAMs to tumor-killing M1 TAMs (**Figure 2**) (12), and we will provide a detailed introduction of nanodrug delivery systems that target TAMs for the treatment of tumors in the following content.

### 2.1 Depletion of M2 TAMs

Macrophages account for 60% of the cellular components in the tumor, and with the progression of the tumor the macrophages in TME are dominated by M2 TAMs which promote tumorigenesis and metastasis, and based on this reducing M2 TAMs in TME can eliminate the pro-tumor effects of macrophages (13). Studies have shown that nanoparticles that respond to overexpression of matrix metalloprotein-2 (MMP-2) by cancer cells can inhibit tumor growth by influencing TAMs in the TME (14). For example, liposomal polyethylene glycol (PEG) - FA Lip delivering immunogenic death (ICD) inducer doxorubicin (DOX) in response to folate modification of MMP-2 can significantly inhibit tumor growth by targeting and depleting M2 type TAMs (15). Moreover, the surface modification of gold nanorods HA-AuNR/M-M2pep with M2-pep fusion peptide in response to MMP2 can release M2-pep in TME with high MMP2 expression and selectively deplete M2-TAMs to improve the immune activity of TME and effectively inhibit tumor growth (16). In addition to MMP-2-responsive nanoparticles that can affect TAMs, colony-stimulating factor 1 receptor (CSF-1R) inhibitors or CSF-1R blockers can also inhibit tumor growth by influencing TAMs, such as nanoparticles loaded with CSF-1R inhibitors can inhibit tumor growth and metastasis by effectively depleting TAMs (12). Nanoparticle A15-BLZ-NPs of the FXIIa substrate peptide A15-modified CSF-1R inhibitor sotuletinib (BLZ945) can remodel and activate the anti-tumor immune response by specifically reducing M2-type TAMs by releasing BLZ945 (17). In addition, a pH-responsive copolymer micelle (dextran grafted poly(histidine) copolymer) camouflaged by erythrocyte-cancer cell hybridization membranes can also deliver the CSF-1R inhibitor BLZ-945 to the TAMs to specifically deplete TAMs and inhibit tumor growth (18). Moreover, dual-targeted nanoparticle M2NPs formed by the addition of a α peptide (scavenger receptor B-type 1 targeting peptide) to an M2 macrophage binding peptide (M2NP) and loading with anti-CSF-1R small interfering RNA (siRNA) can significantly eliminate M2-like TAMs by specifically blocking the survival signal of M2-like TAMs thereby shrinking tumors and prolonging survival (19). In addition, bisphosphonates can also specifically hinder the survival of macrophages (20), such as a calcium bisphosphonite (CaBP-PEG)-based nanoparticle prepared by mineralization with PEG coating can inhibit tumor growth by depleting TAMs as well as reducing angiogenesis (21). Moreover, Biotin and mannose-modified lipid-encapsulated calcium zoledronate nanoparticles CaZOl@BMNP can bind to TAMs and promote zoledronate release thereby reducing TAMs, remodeling the tumor immunosuppressive microenvironment and ultimately inhibiting tumor progression (22). Not only that, “carrier-free” coordination polymer nanorods based on zoledronic acid and gadolinium self-assembly can also specifically deplete TAMs (23). In addition to the nanoparticles being able to affect M2 TAMs in TME mentioned above, inhibition of lactate production can also effectively restore immunosuppressive TME based on the fact that cancer cells continue to produce lactate through aerobic glycolysis thereby promoting TAMs polarization to the M2 phenotype. For example, the catalytic nanosystem composed of erythrocyte membrane (mRBC)-camouflaged hollow MnO_2_ (HMnO_2_) encapsulated lactate oxidase (LOX) and glycolysis inhibitors can deplete lactate in TME and produce oxygen through LOX-catalyzed oxidation of TME, and the combination treatment of tumors with PD-L1 can successfully reverse immunosuppressive in TME by significantly reducing the number of M2 TAMs (24). Moreover, the mannose glycosylated hybrid micellar DAS-MMic that specifically delivers dasatinib (DAS) can also selectively reduce the number of M2-type TAMs thereby reshaping the immunosuppressive tumor microenvironment and ultimately inhibiting tumor progression (25). In conclusion, a variety of nanoparticles have been shown to exert anti-tumor effects by reducing M2-type TAMs thereby reshaping TME, including MMP-2-responsive nanoparticles, nanoparticles loaded with CSF-1R inhibitors or blockers, and bisphosphonate-composed nanoparticles.

### 2.2 Restricting the recruitment and localization of TAMs

Macrophages enter tumors under the action of a variety of chemokines, such as CC motif chemokine 2 (CCL2), CCL3, CCL4, CCL5, colony-stimulating factor-1, and vascular endothelial growth factor (VEGF), based on this the strategy of targeting TAMs to treat tumors can also block TAMs entry into tumors by targeting these chemokines that recruit macrophages (12). For example, gene therapy using siRNA nanoparticles targeting CX3CL1 can reduce the recruitment of Ly6Clo monocytes thereby enhancing the effect of anti-VEGFR2 tumor treatment (26). KLAK-MCP-1 micelles composed of CC motif chemokine receptor-2 (CCR2) targeting peptide sequence (MCP-1 peptide) and apoptotic KLAKLAK peptide can inhibit tumor infiltration of TAMs by blocking the Monocyte Chemoattractant Protein-1 (MCP-1)/CCR2 axis thereby suppressing tumor growth (27). Furthermore, the ultra-small copper nanoparticles Cu@CuOx loaded with CCR2-targeting and the chemotherapy drug gemcitabine can inhibit cancer progression and prolong survival by targeting CCR2 to inhibit TAMs recruitment to tumors by targeting CCR2 (28). In addition, cationic nanoparticles (CNP-siCCR2) encapsulated with siCCR2 can effectively inhibit the expression of CCR2 in monocytes and blocking the recruitment of monocytes to tumor tissues thereby changing the tumor immune microenvironment, inhibiting primary tumor growth and reducing tumor metastasis, and improving the anti-tumor effect of chemotherapy drugs (29). In conclusion, TAMs recruitment and localization can be limited by blocking macrophages entry into tumors by targeting various chemokines targeting macrophages.

### 2.3 Reprogramming of M2 TAMs to M1 TAMs

It is also possible to reprogram the pro-tumor M2 TAMs to the tumor-killing M1 TAMs thereby reversing the immunosuppressive TME to the anti-tumor TME in addition to reducing TAMs in TME and limiting TAMs recruitment and localization (12). A variety of nanodrug delivery systems have been developed to exert anti-tumor effects by modulating TAMs polarization, such as iron-based nanoparticles, nanoparticles loaded with toll-like receptor (TLR) agonists, and nanoparticles loaded with signaling pathway inhibitors or activators (such as phosphatidylinositol 3-kinase γ PI3Kγ inhibitors, c-MYC inhibitors, NF-κB and IRF5 activators) (30, 31), and we will introduce nanoparticles that reprogram M2 TAMs into M1 TAMs to kill tumors in the following content.

#### 2.3.1 Iron-based nanoparticles

A variety of iron-based nanoparticles have been shown to reedit M2 TAMs to M1 TAMs, such as iron in superparamagnetic iron oxide nanoparticles (SPION) that can induce the transformation of human monocytic leukemia cell (THP1)-derived M2 macrophages to a high CD86+, tumor necrosis factor-α (TNF-α) positive M1 macrophage subtype (32). At the same time, the application of iron oxide nanoparticles to treat tumors can also polarize the TAMs in tumor tissue to the M1 phenotype thereby indirectly affecting the TME and inhibiting tumor growth (33). In addition, the ultrasmall nanotrap can capture endogenous iron and target it for transport to the TAMs inside the tumor, and iron was released from the nanotrap when exposed to lysosomal acidity and intracellular H_2_O_2_ and generate oxidative stress thereby reprograming the TAMs to activated M1 TAMs and inducing immune response and ultimately inhibiting tumor growth (34). Moreover, the metal nanoprodrug NPS-G-Fe produced by coordinating Fe^3+^ and gallic acid (GA) with Ce6 in photoactivated Pt(IV) containing nanoprodrug and then coating with PEG-modified chondroitin sulfate (PEG-CS) can lead to iron dissociation under acidic tumor microenvironment conditions, and NPS-G-Fe can promote macrophage polarization to M1 phenotype after radiotherapy thereby enhancing anti-tumor efficacy (35). Furthermore, Fe-MOF nanoparticle MIL88B loaded with ferrodrosis activator RSL3 can stimulate the potent antitumor activity of TAMs (including phagocytic killing and metastatic inhibition) by activating M1-related signaling pathways and downregulating M2 signaling (36, 37). In addition, the nanoparticles MNP@MDSC formed by the coating of zero-valent iron nanoparticles ZVI-NP and magnetic Fe_3_O_4_ nanoparticles (MNP) on MDSCs membranes can also reprogram M2 TAMs to anti-tumor M1 TAMs to enhance anti-tumor immunity (38). In conclusion, a variety of iron-based nanoparticles have been shown to reprogram M2 TAMs to M1 TAMs to exert antitumor activity.

#### 2.3.2 Toll-like receptor agonists

TLR agonists (including TLR7 agonists, TLR-7/8 agonists, and TLR9 agonists) can also polarize M2-like TAMs to M1-like TAMs in addition to iron-based nanoparticles, but their application *in vivo* is limited by the fact that TLRs are easily cleared by nucleases in circulation and can also trigger inflammatory responses through non-specific stimulation (20), while the TLR loaded inside the nanoparticle not only protects the TLR from the effects of nucleases in the blood, reduces the systemic side effects caused by TLR agonists, but also enables them to target TAMs to exert their effects, such as the nanoparticles Poly(L-lactic-co-glycolic acid) (PLGA)- ION-R837@M (PIR@M) formed by the magnetic polymer nanoparticles encapsulated with Fe_3_O_4_ NPs and the TLR7 agonist imiquimod (R837) coated with lipopolysaccharide (LPS)-treated macrophage membranes can target TAMs, in which PIR@M can polarize TAMs from M2 to an anti-tumor M1 phenotype by the synergistic effect of Fe_3_O_4_ NPs and R837 thereby attenuating immunosuppressive TME and activating the anti-tumor immune response (39). In addition to the TLR7 agonist-loaded nanoparticles that can polarize M2 TAMs to M1 TAMs, liposomal R848-LPs loaded with the TLR-7/8 agonist resiquimod (R848) can also rapidly accumulate at tumor sites and R848-targeting TAMs can polarize TAMs to type M1, and this nanoparticle can also enhance the anti-tumor effect of therapeutic antibodies by enhancing antibody-dependent cell phagocytosis (ADCP) effects (40). Moreover, β-cyclodextrin nanoparticles (CDNPs) encapsulated with R848 can also be rapidly absorbed by tumor models and effectively deliver drugs to TAMs *in vivo* and transform TAMs into a tumor-killing M1 phenotype thereby inhibiting tumor growth (41). In addition, R848-loaded mannosan decarboxylated polylactic acid nanoparticles (Man-pD-PLGA-NP@R848) can target TAMs in a mannose receptor-mediated manner and further reprogram TAMs to an anti-tumor phenotype thereby inhibiting tumor progression by reprogramming immunosuppressive TME from “cold tumors” to “hot tumors” (42). In addition, site-directed quantitative coupling of the TLR7/8 agonist imidazoquinolone IMDQ to a single-chain antibody fragment can not only target TAMs by targeting macrophage mannnose receptors (MMRs) on TAMs, but also inhibit tumor growth by promoting TAMs repolarization to a pro-inflammatory phenotype (43). In addition to TLR-7 agonists and TLR-7/8 agonists, M2pep-rHF-CpG nanoparticles formed by the TLR9 agonist CpG oligonucleotide (CpG ODN) are enclosed in human ferritin heavy chain (rHF) nanocage engineered by mouse M2 macrophages, these nanoparticles can be targeted to deliver to M2 TAMs and polarize M2 TAMs into M1 TAMs thereby inhibiting tumor growth (44). In conclusion, a variety of nanoparticles using nanomaterials to deliver TLR agonists have been shown to polarize M2 TAMs to M1 TAMs in TME to exert anti-tumor efficacy.

#### 2.3.3 Pathway inhibitors or activators

In addition to iron and TLR agonist-based nanoparticles, some pathway inhibitors or activators can also target TAMs and polarize M2-type TAMs to M1-type TAMs, such as PI3Kγ inhibitors, c-MYC inhibitors, NF-κB and IRF5 activators. Among them, the PTEN/PI3Kγ/mTOR signaling pathway has been shown to be involved in macrophage repolarization, such as porous hollow iron oxide nanoparticles PHNP@3-MA loaded with P13Kγ small molecule inhibitors modified with mannose can target TAMs, and PHNPs combined with 3-MA can activate the inflammatory factor NF-κB p65 of macrophages thereby converting TAMs into pro-inflammatory M1 macrophages, activating immune responses and inhibiting tumor growth *in vivo (*45, 46). The CSF-1/CSF-1R pathway is also involved in the infiltration and polarization of immunosuppressive cells in addition to the PI3K-γ pathway, based on this the modification of the M2 TAMs targeting peptide M2pep on the nanoparticles co-encapsulated with the PI3K-γ inhibitors NVP-BEZ 235 and CSF-1R-siRNA can not only co-deliver PI3k-γ and CSF-1R to M2 TAMs but also reshape the inhibitory tumor microenvironment by decreasing the level of M2-TAMs and increasing the level of M1-TAMs compared with single-path blockade, and can activate anti-tumor immune responses thereby enhancing anti-tumor effects (47). Moreover, simultaneous inhibition of CSF1-R and the Src homology region 2 (SH2) domain phosphatase SHP-2 signaling pathway in macrophages can also effectively revert M2 macrophages to the M1 phenotype and enhance the phagocytosis of macrophages, for example, self-assembled nanoparticles (DNTs) loaded with dual inhibitors of CSF1R- and SHP2 can target M2 macrophages and cause M2 macrophages to repolarize to the M1 phenotype by simultaneously inhibiting the CSF1R and SHP2 pathways, and which has stronger phagocytosis compared to drug therapy alone, showing enhanced antitumor effects (48). In addition to pathway inhibitors, some pathway activators can also promote the reprogramming of M2 TAMs to anti-tumor M1 TAMs, such as nanoparticles Gd@C82 modified with β-alanine (GF-Ala) can be highly internalized by TAMs and reprogram TAMs from tumor-promoting M2 phenotype to tumor-killing M1 phenotype by activating NF-κB and IRF5 pathways thereby remodeling immunosuppressive TME, triggering effective anti-tumor immunity and effectively inhibiting tumor growth (49). At the same time, transcription factors such as c-MYC can also control the inflammatory response of macrophages and polarize them towards the M2 phenotype whereas c-MYC inhibitors can block this effect (50). For example, perfluorocarbon nanoparticles encapsulated with MYC inhibitor prodrug MI3-PD can be incorporated into M2 tumor-promoting macrophages through phagocytosis-induced and dependent mechanisms thereby significantly reducing proto-cancer M2-like macrophages while retaining anti-tumor M1-like macrophages (51). In addition, CCL2 and CCL5 are considered to be the two main chemokines of TAMs, which responsible for attracting TAMs infiltration and inducing its polarization towards the oncogenic M2 phenotype whereas blocking CCL2 and CCL5 can block the occurrence of this phenomenon (52, 53), such as the mRNA encoding BisCCL2/5i encapsulated in a lipid nanoparticle platform can block both CCL2 and CCL5 signaling thereby significantly inducing TAMs to the anti-tumor M1 phenotype polarization and reversing immunosuppression in the TME (54). In conclusion, a variety of nanoparticles encapsulating pathway inhibitors or activators have been shown to exert anti-tumor immune responses to inhibit tumor growth by targeting TAMs and reprogramming M2 TAMs into M1 TAMs in the TME.

#### 2.3.4 Other nanoparticles that reprogram TAMs

In addition to TLR agonists and signaling pathway inhibitors or activators, there are various other nanoparticles that have been shown to exert anti-tumor effects by targeting TAMs and reprogramming M2 TAMs into M1 TAMs. For example, biomineralized nanoparticles SBC@CaP derived from aging red blood cell vesicles and coated with pH-responsive calcium phosphate (CaP) shells, gradually dissolve the CaP layer in the acidic TME and expose the aging red blood cell membrane, enabling TAMs to selectively recognize and phagocytize them, achieving precise TAMs clearance, TME remodeling and immune activation thereby inhibiting tumor progression (55). PLGA nanoparticles coated with baicalin and the melanoma antigen Hgp peptide fragment 25-33, prepared using ultrasound double-emulsion technology and further loaded with CpG fragments and forming a nanocomposite on their surface with conjugated M2pep and α-pep peptides, can be effectively taken up by M2 TAMs and convert M2-like TAMs into M1-like phenotypes thereby remodeling the TME and killing tumor cells (56). In addition, a near-infrared II (NIR-II) fluorescence/photoacoustic nano-inducer I/E@M2pep which can be activated by nitric oxide (NO), where the M2pep peptide can target M2 TAMs and IPI549 reprograms them into an M1 phenotype, and can significantly enhance antitumor immunity through M1 TAM-mediated tumor killing and TME remodeling when combined with a CD47 monoclonal antibody (57). X-ray-responsive iron-ethylene glycol-chitosan-polypyrrole nanoenzymes GCS-I-PPy NZs can also be internalized by M1 TAMs and activate them, enhancing immune responses and adaptive immunity (58). Moreover, a caspase B (CTSB)-responsive programmed targeted delivery system D&R-HM-MCA can achieve TAM-targeted delivery, and the outer angiopep-2 modification can be cleaved off by CTSB-responsive peptides when it reached the tumor site, exposing the para-aminophenyl-α-d-mannopyranoside (MAN) modification which can further recognize glucose transporter-1 (GLUT1) on tumor cells and macrophage mannose receptors (MMR) on TAMs, inducing TAMs polarization from the anti-inflammatory M2 phenotype to the pro-inflammatory M1 phenotype thereby improving antitumor immune responses (59). In addition, polymeric poly(styrene-comaleic anhydride) (PSMA)-NPs and conjugated polymer poly[2-methoxy-5-(2-ethylhexoxy)-1,4-styreneyl](PPV) -PSMA-NPs nanoparticles prepared by nanoprecipitation have also been shown to reverse the immunosuppressive TME by reprogramming TAMs, and which can not only specifically target TAMs but also directly inhibit tumor growth by repolarizing TAMs to kill tumor M1 phenotype (60). In summary, nanoparticles made from various materials have also been proven to exert anti-tumor immunity by targeting TAMs and reprogramming them.

In addition to the nanoparticles mentioned above that exert antitumor effects by targeting and reprogramming TAMs, there are also many nanoparticles that may reprogram them through non-targeted TAMs. For example, the nanoparticle NanoMnSor composed of a MnO_2_ core and lipid and loaded PLGA with sorafenib can co-deliver sorafenib and MnO_2,_ where MnO_2_ can reduce hypoxia by reacting with H_2_O_2_ produced by overactive metabolism of tumor cells and H^+^ in acidic TME to produce oxygen, which can not only overcome resistance to sorafenib but also promote macrophage polarization towards the immune-stimulated M1 phenotype (61). At the same time, calcium carbonate nanoparticles loaded with anti-CD47 antibodies encapsulated in fibrin gels and PLGA nanoparticles that encapsulate baicalin and melanoma antigen Hgp peptide fragments and further encapsulate CpG fragments with conjugated M2pep and α-pep peptides on the surface all can repolarize tumor-promoting M2 macrophages into tumor-killing M1-type macrophages (56, 62). In addition, lipid nanoliposomes composed of vitamin E and sphingomyelin (VitE: SM) can reduce the expression of the macrophage M2 markers Arg1 and Egr2, and increase the expression of the M1 markers Cd86, Il-1b, and Il-12b, that is converting the M2 phenotype to M0/M1 state (63). Not only that, HA-DEX-DOX nanoparticles composed of micelles containing the chemotherapeutic drug doxorubicin, selenium nanoparticles (SeNPs) and CS-I/J@CM NPs nanoparticles composed of Cu2-xSe, indoxime (IND, an inhibitor of indolamine-2,3-dioxygenase in tumors), JQ1 (PD-L1 inhibitor) and tumor cell membranes can all repolarize M2 macrophages into M1 macrophages thereby exerting immunomodulatory effects to inhibit tumor growth (64–66). Besides, tumor-associated macrophage membranes (TAMMs) derived from primary tumors can block the interaction between TAMs and cancer cells by depleting CSF1 secreted by tumor cells in the TME, such as NPR@TAMM nanoparticle-mediated photodynamic immunotherapy formed by TAMM coated with conjugated photosensitizers can convert macrophages from an immunosuppressed M2-like phenotype to a more inflammatory M1-like phenotype thereby improving the anti-tumor immune response (67). Similarly, nanoparticle NK NPs loaded with photosensitizers 4,4’,4’’,4’’’-(porphine-5,10,15,20-tetrayl) tetrakis (benzoic acid) (TCPP) encapsulated by NK cell membranes can also target tumors and induce pro-inflammatory M1-macrophage polarization thereby generating anti-tumor immunity to eliminate primary tumors and inhibit distant tumors (68). A FePt-based biomimetic nanocapsule FP/Vad@CC-aT2 modified with triggering receptor 2 (TREM2) expressed on myeloid cells for delivering the STING agonist Vadimezan (Vad), where anti-TREM2 can effectively repolarize TAMs into M1 macrophages thereby reversing the immunosuppressive TME together with the Vad-activated STING pathway and inhibiting tumor recurrence (69). In conclusion, the repolarization of tumor-promoting M2 TAMs to M1-type TAMs that kills tumors is a major strategy to reverse immunosuppressive TME to anti-tumor TME by influencing TAMs, and a variety of nanodrug delivery systems have been developed, such as iron-based nanoparticles, nanoparticles loaded with TLR agonists, and nanoparticles loaded with signaling pathway inhibitors or activators.

## 3 Nanoparticles regulate NK cells

**Figure 3:**
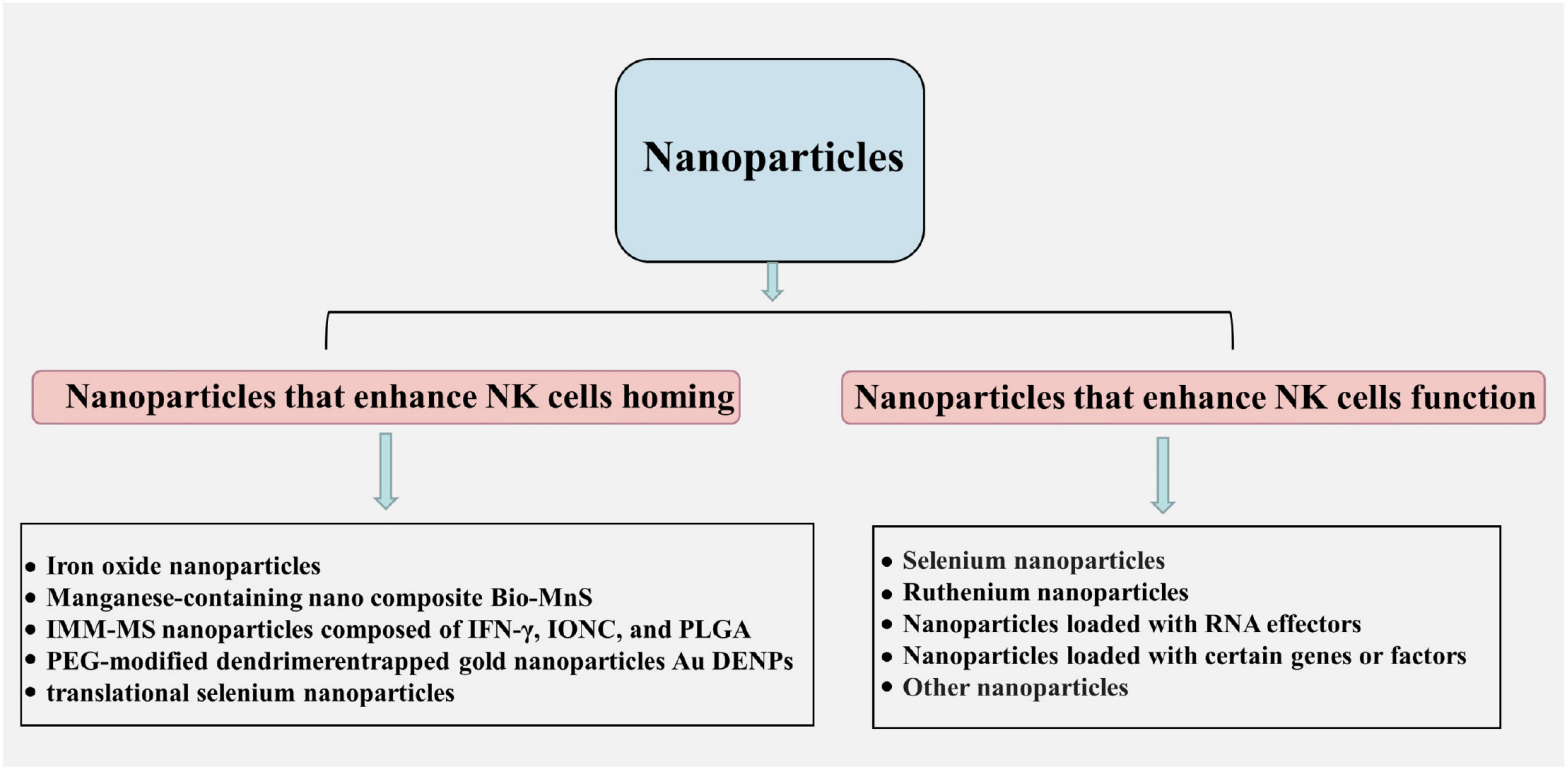
Nanoparticles that regulate NK cells to enhance antitumor immunity: including promoting NK cells homing (including iron oxide nanoparticles, manganese-containing nano composite Bio-MnS, IMM-MS nanoparticles composed of interferon-γ, IONC and PLGA, PEG-modified dendrimer-encapsulated gold nanoparticles Au DENPs, transformation-type selenium nanoparticles, etal.) and enhancing NK cell function (including selenium nanoparticles, ruthenium nanoparticles, nanoparticles loaded with RNA effectors, nanoparticles loaded with certain genes or factors, and other nanoparticles).

NK cells in tumors are responsible for destroying tumor cells and preventing tumor development and progression, and activated NK cells can not only produce direct cytotoxic effects through perforin, granzyme release, or death receptor signaling, but also produce cytokines and chemokines to regulate other parts of the immune response (70). However, NK cells activation is greatly inhibited due to the overproduction of transforming growth factor β (TGF-β) and other anti-inflammatory cytokines and chemokines in the TME (71, 72). It has been found that many nanoparticles can play an anti-tumor role by modulating NK cells to enhance anti-tumor immunity with the application of nanotechnology in tumor therapy, and common strategies to modulate NK cells include promoting NK cells homing and enhancing NK cells activity (**Figure 3**) (73).

### 3.1 Nanoparticles enhance NK cells homing

A variety of nanoparticles have been shown to promote NK cells homing, such as iron oxide nanoparticles bound to the surface of primary NK cells can not only significantly enhance NK cells homing to tumors but also show significant anti-tumor efficacy by increasing the expression of granzyme and perforin in tumor cells compared to unmodified NK cells (73). In addition, compared with pure selenium nanoparticles and free manganese, a manganese-containing nanocomplex Bio-MnS synthesized by chlorella as a bioreactor can promote the infiltration of NK cells into tumors by releasing a large amount of interferon thereby achieving anti-tumor immunity (74). At the same time, IMM-MS nanoparticles composed of recombinant interferon γ (IFN-γ), iron oxide nanotubes (IONC) and PLGA were successfully prepared by the double-emulsion method, which can not only be deposited in liver tumors but also continuously release IFN-γ and increase the infiltration of NK cells into tumor sites (75). In addition, PEG-modified dendritic dimethylcyclogold nanoparticles (Au DENPs) can carry NK cells transfected with the human ferritin heavy chain (hFTH1) gene and guide them to gather around tumors for effective gene therapy thereby killing cancer cells (76). Besides, SeNPs have been shown to promote NK cells proliferation and enhance their cytotoxicity against cancer cells by activating the GPX-driven mTOR signaling pathway, and enhance the anti-tumor therapeutic effect when combined with chemotherapy (77). In conclusion, a variety of nanoparticles have been shown to exert anti-tumor efficacy by promoting NK cells homing or proliferation.

### 3.2 Nanoparticles enhance NK cells function

Nanoparticles can also exert anti-tumor immunity by enhancing NK cells function in addition to promoting the homing of NK cells to the tumor site, such as SeNPs designed with neutral (polyvinylpyrrolidone-PVP), anionic (citric acid-LET) and cationic (chitosan-CS) surfactants, which can not only trigger more NK cells in mouse but also effectively activate NK cells (78). Moreover, the selenium peptide nanoparticles SeP/DOX loaded with DOX can not only increase their accumulation in tumors and rapidly release loaded drugs by identifying αvβ3 integrins overexpressed on the surface of endothelial cells in tumors and tumor neovasculature, but also activate the immune response of NK cells through oxidative metabolites such as alkyl selenic acid to promote the improvement of anti-tumor efficacy (79). In addition, PEGylated hollow mesoporous ruthenium nanoparticles as a carrier to load the fluorescent anti-tumor complex ([Ru(bpy)2(tip)]2+, RBT) and a conjugate with bispecific antibodies (SS-Fc) can promote NK cells interaction with tumor cells and further trigger NK cell-mediated target cells lysis (80). Not only that, ruthenium (Ru) dopyridine complex RuPOP can promote NK cells infiltration and interaction with tumor cells by upregulating natural killer cell group 2D (NKG2D) and its ligands thereby showing high anti-tumor efficacy (81). In addition to selenium nanoparticles and ruthenium nanoparticles, nanoparticles loaded with RNA effectors can also exert anti-tumor immunity by enhancing NK cell activity, such as siRNA, microRNA (miRNA), and short hairpin RNA (shRNA) can enhance antitumor activity by silencing specific genes to alter genomic function (82). For example, TGF-β receptor-2 (TGFBR2) can inhibit NK cells function while nanoparticles carrying TGFBR2 siRNA can enhance the anti-tumor activity of NK cells by protecting NK cells from immunosuppression by inhibiting TGFBR2 (75). Moreover, the non-viral lipid nanodelivery system that encapsulates siRNA can also target NK cells *in vivo* and silence the inhibitory checkpoint signaling molecules SHP-1, Cbl-b, and c-Cbl of NK cells, thereby enhancing the killing activity of NK cells against cancer cells and eliminating tumor (83). In addition to this, EpCAM (epithelial cell adhesion molecule)-targeted cationic liposome (LPP-P4-Ep) containing si-CD47 and si-PD-L1 can not only effectively silencing CD47 and PD-L1 but also increases the percentage of NK cells to promote NK cells responses (84). Furthermore, the encapsulation of siCD155 in cationic lipid nanoparticles (CLANsiCD155) can effectively deliver siCD155 into infiltrating macrophages and tumor cells, and downregulate CD155 thereby increasing NK cell activation and inhibiting tumor proliferation (85). Not only that, the tumor suppressor gene (TUSC2) encoding a multi-kinase inhibitor delivered through nanovesicles can not only increase circulating and spleen NK cells but also significantly enhance anti-tumor activity and immune response by activating NK cells in combination with anti-PD-1 therapy (86). In conclusion, selenium nanoparticles, ruthenium nanoparticles, and various nanoparticles carrying RNA effectors can all exert antitumor effects by enhancing NK cell activity.

Nanoparticles loaded with certain genes or factors can also exert anti-tumor effects by activating NK cells (73). For example, chitosan-based PcDNA3.1-dsNKG2D-IL-21 plasmid nanoparticles were prepared by linking a two-gene fragment encoding NKG2D and the extracellular domain of interleukin (IL)-21 gene and inserting into the pcDNA3.1 plasmid, which can delay tumor growth by activating NK cells *in vivo* after tumor tissue accumulation (87). Moreover, chitosan nanoparticles loaded with dsNKG2D-IL-15 fusion gene fragments can also deliver IL-15 and NKG2D genes into cancer cells and bind to NKG2D receptors in NK cells to activate NK cells thereby inducing anti-tumor immune responses and inhibiting tumor growth and ultimately prolonging the survival of tumor-bearing mouse (88). In addition, cyclic diGMP (c-di-GMP) is considered to be an anti-cancer adjuvant and which is a ligand for the interferon gene stimulator (STING) signaling pathway, when it was encapsulated in YSK05 liposome to form c-di-GMP/YSK05-Lip nanoparticles which can effectively induce the production of type I IFN and activate NK cells in mouse, and induce NK cell-mediated MHC-I non-restricted anti-tumor immunity to produce significant anti-tumor effects (89). In addition, many nanoparticles have also been shown to exert anti-tumor effects by enhancing NK cells activity, such as cationic nanoparticle cNPs can effectively inhibit tumor growth by altering the expression of chemokine receptor CCR4 and C-X-C motif chemokine receptor-4 (CXCR4) in NK cells and activating NK cells (90). Hydroxyapatite (HAp)-modified chitosan (CS) thermosensitive hydrogel-formed biomaterial complexes can also enhance anti-tumor effects by prolonging the retention time of NK cells in tumors and improving NK cells function (91). Moreover, the multifunctional NK cells nanoconjugate FBS-Bru can promote tumor-responsive release of divalent iron ions (Fe^2+^) and brusatol (Bru), significantly enhancing the infiltration and activation of NK cells within tumors thereby improving tumor-specific cytolytic efficacy (92). Meanwhile, HEFDS composed of magnetic nanoparticles modified with cationic polyethyleneimine (PEI), selenocysteine (Sec), and sodium hyaluronate (HA), can form HEFDS-NK cells through co-culture with NK cells and can reach tumor sites with the assistance of HA and magnetic field targeting, where Sec can enhance the immune activity of NK cells, producing Granzyme B, Perforin, and IFN-γ, ultimately achieving effective tumor treatment (93). In addition, nanoemulsions SSB-NMs co-delivered with TGF-β inhibitors and selenocysteine (SeC) can effectively inhibit TGF-β/TGF-β RI/Smad2/3 signaling thereby stimulating the expression of NKC2D on NK92 cells and NKG2DL on tumor cells and significantly improving the lytic capacity of NK92 cells and promoting the immune response (94). Not only that, multifunctional nanoparticles MF-NPs with core-shell structure synthesized from cationic polymers labeled with NIR fluorescent molecules combined with polydopamine (PDA) coatings and the superparamagnetic nanoparticle ZCMF formed by the core-shell structure modified by an anti-VEGF antibody and Zn^2+^-doped Zn-CoFe_2_O_4_@Zn-MnFe_2_O_4_ all can enhance NK cell-mediated antitumor activity (95, 96). In conclusion, a variety of nanoparticles have been found to exert anti-tumor effects by activating NK cells, including selenium nanoparticles, ruthenium nanoparticles, and nanoparticles loaded with certain RNAs, genes, or factors.

## 4 Nanoparticles regulate DCs

DCs are specialized antigen-presenting cells that can capture the antigens released by tumor cells and present them to T cells in tumor-draining lymph nodes to produce tumor-specific cytotoxic T lymphocytes (CTLs) (97), besides DCs can also stimulate NK cells and B cells to activate humoral immunity (98), therefore, strategies that increase the number of DCs or promote their maturation can enhance anti-tumor immunity. At present, it has been found that a variety of nanoparticles can exert anti-tumor efficacy by promoting DCs recruitment or maturation. For example, nanodiscs sHDL composed of phospholipids and apolipoprotein-1 mimetic peptide (22A) that co-deliver antigen (Ag) peptides and adjuvants can efficiently co-deliver Ag and CpG to draining lymph nodes, promote DCs antigen presentation, induce DCs maturation and trigger strong antitumor effects (99). In addition, the biomimetic cancer cell membrane (EPBM) engineered to express peptides, encapsulated in the nanovaccine drug delivery system PLGA/STING@EPBM, can deliver STING agonists and tumor antigens to Clec9a DCs, significantly enhance IFN-induced gene expression and antigen cross-presentation in Clec9a DCs, and exhibit strong antitumor effects (100). At the same time, a poly(lactic-co-glycolic acid) (PLGA) nanovaccine co-delivering the adjuvant R837 and Luc-4T1 cell membrane antigen, with surface-exposed calreticulin, can induce active uptake by DCs thereby enhancing the anti-tumor effect (101). A nanovaccine formulated using pH-responsive amphiphilic diblock copolymers synthesized via reversible addition-fragmentation chain transfer (RAFT) polymerization, conjugated with STING agonist ADU-S100 and mannose as well as further combined with antigen peptides and polyethyleneimine, can effectively inhibit tumor growth by specifically targeting DCs and efficiently activating them to promote antigen presentation (102). In addition to the nanoparticles mentioned above that target and activate DCs, there are also nanoparticles based on polysaccharides and other materials that may exert anti-tumor effects by non-targeting DCs or with unclear targeting but still activate DCs. For example, encapsulating the adjuvant monophosphoryl lipid A in the core of poly(D, L-lactide-co-glycolide) nanoparticles and then modifying the surface with the tumor-targeting peptide TMTP1 and the DC receptor mannose to form the nanovaccine NP-TP1@MM can capture and enrich more tumor-specific antigens after chemotherapy, stimulate DCs maturation and activate adaptive immunity (103). In addition, a therapeutic nanovaccine (NV) mediated by CuS-R848 coated with HA can significantly inhibit tumors and prevent tumor recurrence and metastasis by inducing immunogenic cell death (ICD) and DCs maturation (104). Moreover, a self-adjuvanting gel (SAG) formed by grafting 4-benzimidazole-modified polyethyleneimine (PEI-4BImi) with oxidized dextran which has natural immune-activating properties, can inhibit tumor growth by delivering doxorubicin and the TLR-9 agonist CpG to tumor sites and lymph nodes, promoting antigen presentation and enhancing DC activation within lymph nodes (105). Besides polysaccharide-based nanoparticles, nanoparticles made of other materials can also activate DCs, such as RNA-lipid complex (RNA-LPX) can precisely and effectively target DCs *in vivo*, where LPX protects RNA from extracellular ribonucleases and can mediate the efficient uptake and expression of DCs to encoded antigens, triggering plasmacytoid DCs and activating DCs *in situ* maturation (106). In addition, the nanopreparations CPS/HPPH/DOX loaded with the chemotherapy drug DOX and the photosensitizer 2-(1-hexyloxyethyl)-2-devinyl pyropheophorbide-a (HPPH) can not only recruit DCs to trigger the immune cascade but also increase the mature DCs in the tumor draining lymph nodes to inhibit primary and distant tumor growth (107). Moreover, calcium carbonate nanoparticles MC/Dox/Ce6 are coated by a multifunctional cancer cell membrane loaded with Dox and photosensitizer chlorine e6 can also elicit tumor-associated antigens (TAAs) and recruit DCs, and the recruited DCs can be activated *in situ* to effectively inhibit cancer growth (108). Furthermore, polydopamine (PDA) nanoparticles loaded with Ce6 molecules can be synergized with phototherapy to release antigens thereby triggering DCs maturation and helping to inhibit tumor metastasis and recurrent (109). Not only that, the CMS/Au heterostructure constructed by depositing plasma Au nanoparticles onto Cu_2_MoS_4_(CMS) nanosheets can eradicate primary and metastatic tumors by promoting DCs maturation and secreting cytokines under NIR laser irradiation (110). In conclusion, a variety of nanoparticles have been shown to exert anti-tumor efficacy by promoting DCs recruitment or maturation.

## 5 Nanoparticles regulate TANs

Neutrophils in the bone marrow are released and migrate to the tumor microenvironment under the stimulation of mediators such as granulocyte colony-stimulating factor (G-CSF), granulocyte-macrophage colony-stimulating factor (GM-CSF), and chemokines such as CXC and CCL3 (111), and TANs in TME exhibit two subtypes under the action of cytokines: N1 TANs with antitumor effect and N2 TANs with tumor supportive activity. Based on this neutrophils can also be used as targets for anticancer therapy (5), the main strategies include reducing neutrophils in tumor sites by inhibiting neutrophil recruitment and migration, enhancing the antitumor activity of neutrophils, and altering neutrophil polarity. Until now, various nanoparticles have been shown to inhibit tumor growth by influencing TANs, for example, DOX-loaded micellar low molecular weight heparin-aspartate-asparagine nanoparticles (LMWH-AST/DOX, LA/DOX-NP) can close the inflammatory feedback and the formation of neutrophil extracellular traps, alleviate the tumor immunosuppressive microenvironment thereby inhibiting tumor metastasis (112). Moreover, mP-NPs-DNase/PTX nanoparticles consisting of a paclitaxel (PTX) prodrug nanoparticle core and a poly-l-lysine (PLL) conjugated with the matrix metalloproteinase 9 (MMP-9)-cleavable Tat-peptide-coupled deoxyribonuclease I (DNase I) shell can inhibit malignant tumor growth and distant metastasis by releasing DNase I responsive MMP-9 and further degrading the structure of tumor associated neutrophil extracellular traps after accumulation at the tumor site (113). In addition to reducing neutrophils at tumor sites, anti-tumor effects can also be achieved by altering neutrophil polarity. For example, siTGFβ-PLP-Nesn anoparticles composed of neutrophils (NEs) and cationic liposomes carrying paclitaxel/TGF-β (TGF-β) siRNA, can also target tumor sites and prevent tumor-associated neutrophils from transitioning from an anti-tumor (N1) phenotype to a pro-tumor (N2) phenotype thereby enhancing anti-tumor immunity and inhibiting tumor growth (114). Furthermore, dual-targeting nanovaccines composed of heterocyclic lipids (A18) and polyesters (BR647) simultaneously incorporating oligomeric hyaluronic acid and DMG-PEG2000-mannose and encapsulating STAT3 siRNA and model antigens, can activate the STING pathway and promote the polarization of tumor-associated neutrophils from N2 to N1 (115). In addition, inhalation of virus-like nanoparticles self-assembled from cowpea mosaic virus (CPMV) can be rapidly taken up and activated by neutrophils in the TME, and activated and tumor-infiltrating (N1) neutrophils significantly increased 24 hours after inhalation of eCPMV, ultimately delaying tumor progression and significantly prolonging survival (116). In summary, various nanoparticles have been shown to inhibit tumor growth by influencing TANs.

## 6 Nanoparticles regulate MDSCs

**Figure 4:**
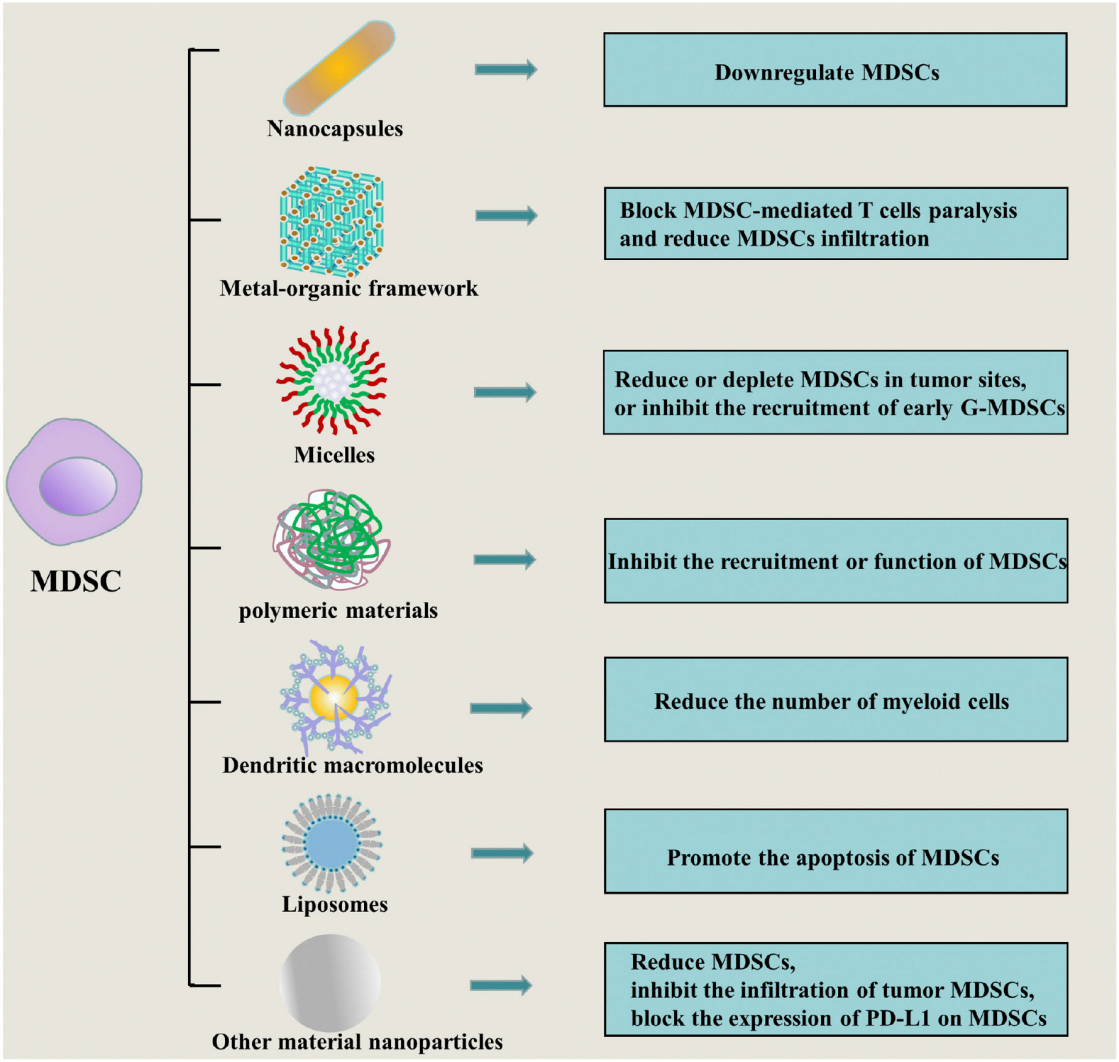
Nanoparticles that regulate anti-tumor immunity by affecting MDSCs: including nanocapsules (by downregulating MDSCs), metal-organic frameworks (by blocking MDSC-mediated T cell paralysis and reducing MDSCs infiltration), micelles (by reducing or clearing MDSCs at the tumor site, or inhibiting the recruitment of early G-MDSCs), polymeric materials (by inhibiting the recruitment or function of MDSCs), dendritic macromolecules (by reducing the number of myeloid cells), liposomes (by promoting MDSCs apoptosis), and other material nanoparticles (by reducing MDSCs, inhibiting the infiltration of tumor MDSCs, blocking PD-L1 expression on MDSCs, etc.).

MDSCs are a heterogeneous population of immature myeloid cells, which play a vital role in promoting tumor progression, metastasis, and the production of immunosuppressive TME, and are associated with treatment resistance and poor prognosis of malignant tumors, and the elimination of immunosuppression of MDSCs is beneficial to restore anti-tumor immunity (117, 118). Nanoparticles composed of various nanomaterials have been shown to regulate anti-tumor immunity by reducing circulating and tumor-infiltrating MDSCs, altering MDSC phenotypes, or inhibiting MDSCs functions, such as nanocapsules, metal-organic frameworks (MOFs)-based nanoparticles, polymeric micelles, polymers, dendritic macromolecules, liposomes, etc (**Figure 4**), and we will introduce the nanoparticles that affect MDSCs in detail in the following content.

### 6.1 Nanocapsules and MOFs

Multilayer polymer nanocapsules are potential nanoparticles for the regulation of MDSCs, in which multilayer polymer nanocapsules encapsulating the chemokine CCL2 of mononuclear macrophage MDSCs and RNAi sequences shC/EBPβ and miR 142-3p that regulate the CCAAT/enhancer-binding protein β (C/EBPβ) pathway covered by polyarginine and hyaluronic acid layers can be RNAi transfected in MDSCs, down-regulate C/EBPα in spleen and tumor MDSCs thereby reversing the immunosuppressive phenotype and are potential nanoparticles for modulating MDSCs (119). Moreover, lipid nanocapsules (LNCs) loaded with load-laurylated gemcitabine (GemC12) can effectively target the monocytic (M-) MDSC subset, and subcutaneous injection of GemC12-loaded LNCs can enhance tumor immunotherapy by reducing the proportion of M-MDSCs in splenic and tumor-infiltrating monocytes, with efficacy higher than that of free gemcitabine (120). Not only that, a graphite nanocapsule formed by nitrogen-doped carbon nanotube cups (NCNC)effectively plugged by chloroauric acid to reduce sodium citrate by gold nanoparticles can effectively deliver paclitaxel to tumor-associated MDSCs and significantly downregulate the expression of TGF-β in MDSCs, which not only inhibited the immunosuppressive phenotype of MDSCs but also differentiated them into DCs thereby reversing their immunosuppressive activity (121). MOF-based nanoplatforms can also modulate anti-tumor immunity by influencing MDSCs in addition to nanocapsules, such as the (M+H)@ZIF/HA nanoparticles formed by the zeolite imidazolate framework-8 (ZIF-8) of the hyaluronic acid-modified mitoxantrone (MIT) and the DNA demethylating agent hydralazine (HYD) with pH response can convert cold tumors into antigen repertoire, stimulate robust immune responses and can effectively block MDSC-mediated T cell paralysis, triggering a strong cytotoxic T cell response to eliminate tumors (122). In addition, the HA/ZIF-8@Gem/D-1-MT nanoparticles obtained by loading gemcitabine and D-1-methyltryptophan into ZIF-8 and then modifying with HA, can also reactivate anti-tumor immunity by inhibiting indoleamine 2,3-dioxygenase and reducing the infiltration of M-MDSCs and G-MDSCs (123). In summary, various nanocapsules and MOF-based nanoparticles have been shown to reverse the immunosuppressive tumor microenvironment either by reversing or inhibiting the immunosuppressive phenotype of MDSCs or by reducing the proportion of tumor-infiltrating MDSCs.

### 6.2 Micelles and polymers

In addition to nanocapsules and metal-organic frameworks, various nanoparticles composed of micelles and polymers have also been shown to modulate anti-tumor immunity by inhibiting the recruitment of MDSCs or directly depleting tumor-infiltrating MDSCs. For example, the pH-sensitive conjugated micelle system (PAH/RGX-104@PDM/PTX) that encapsulates the hepatic X-nuclear receptor (LXR) agonist RGX-104 and paclitaxel not only has excellent tumor accumulation and tumor penetration but also reduces the level of immunosuppressive MDSCs and significantly reduces tumor immunosuppression, enhances the anti-tumor effect of CTLs thereby inhibiting tumor growth *in vivo (*124). In addition, ultra-small polymeric micelles loaded with 6-thioguanine (MC-TG) can not only reduce the number of circulating mononuclear MDSCs (Mo-MDSCs) and granulocyte MDSCs (G-MDSCs) but also deplete MDSCs in the TME, and which have anti-tumor immunity-promoting effects when used in combination with T-cell immunotherapy (125). Moreover, the hydrophilic segment LMWH in a micellar low-toxicity low-molecular-weight heparin-tocopheryl succinate nanoparticle (LMWH-TOS nanoparticle, LT-NP) can inhibit early lung recruitment of G-MDSCs by blocking G-MDSCs extravasation by inhibiting P-selectin/PSGL-1-mediated adhesion between vascular endothelial cells and G-MDSCs, and the hydrophobic segment (TOS) in LT NPs can significantly inhibit the expression of MMP-9 in G-MDSCs thereby maintaining the normal microenvironment of the lungs and effectively inhibiting the implantation and colonization of circulating tumor cells (CTCs) (126). In addition, biomimetic NM/PPcDG/D nanosystems formed by self-assembly and cross-linking of redox-responsive polymers (PPDG) and DOX loaded in nanonuclei (PPcDG/D) then coated with activated neutrophil membranes can inhibit the recruitment and function of MDSCs thereby alleviating MDSC-mediated immunosuppression and inhibiting the formation of lung premetastatic niches (PMNs) and lung metastatic (127). Moreover, the redox reaction nanoassembly R-mPDV/PDV/DOX/sil formed by self-assembly of three glutathione responsive polymers can be broken down by GSH-induced cleavage of 3, 3’-dithiodipropionic acid (DA) to achieve abrupt drug release and efficient lactate dehydrogenase A (LDHA) silencing thereby inhibiting G-CSF and GM-CSF production and further inhibiting MDSCs recruitment and enhancing anti-tumor immunity (128). In conclusion, nanoparticles composed of micelles and polymers can also regulate anti-tumor immunity by inhibiting the recruitment of MDSCs or directly depleting tumor-infiltrating MDSCs.

### 6.3 Dendritic macromolecules, liposomes, and other material nanoparticles

Chemotherapy drugs compounded with dendritic macromolecules can also help eliminate tumor-induced myeloid cells in addition to the previously mentioned nanoparticles. For example, combining anti-Flt1 antibodies loaded with gemcitabine with PEG-polyamidoamine (PAMAM) dendrimer macromolecule complexes in tumor-bearing mouse models can significantly reduce the number of myeloid cells and enhance the efficacy of gemcitabine in treating tumors (129). In addition, IPI-549 transported in liposomes encapsulating the PI3Kγ inhibitors IPI-549 and Ce6 can inhibit PI3Kγ in MDSCs resulting in the downregulation of arginase 1 (Arg-1) and reactive oxygen species (ROS) thereby promoting the apoptosis of MDSCs and reducing their immunosuppression of CD8^+^ T cells, significantly inhibiting tumor growth (130). In addition, various other nanoparticles have also been shown to improve the immunosuppressive tumor microenvironment by reducing MDSCs infiltration or decreasing MDSCs activity, such as high-density lipoprotein-like nanoparticles (HDL-NP) can specifically target the high-affinity receptor B-1 scavenger receptor (SCARB1) of spherical high-density lipoprotein (HDL) expressed by MDSCs thereby inhibiting MDSCs activity and enhancing the immune response to cancer and significantly inhibiting tumor growth (131). Not only that, synthetic protein nanoparticles (SPNPs) coated with the transcellular peptide iRGD (AMD3100-SPNPs) can reduce CXCR4+ M-MDSCs infiltration into TME by blocking C-X-C motif chemokine ligand-12 (CXCL12)/CXCR4 signaling, and combined radiation therapy can lead to long-term survival in mouse (132). At the same time, nanoprodrugs (FIT nanoparticles) that deliver tadalafil (TAD) and indocyanine green (ICG) photosensitizers can not only achieve the controlled release of TAD and ICG at tumor sites but also effectively improve the immunosuppression of MDSCs thereby enhancing the immune response (133). In addition, carrier-free nanoparticles GEM-CXB NPs formed by the nano self-assembly of the dual drugs gemcitabine and celecoxib, exhibit dual clearance of MDSCs and tumor cells thereby significantly enhancing antitumor effects by inducing ICD and alleviating MDSC-induced immunosuppression through MDSCs clearance (134). The integration of a polyphenol-vanadium system with the EZH2 degrader YM281 proteasome-targeting chimeric (PROTAC), encapsulated in methoxy polyethylene glycol-NH2 to form a novel multifunctional nano PROTAC (YM@VBM), can activate antitumor immunity by reducing MDSCs and inhibit tumor growth (135). Meanwhile, a semiconductor polymer nanoscale PROTAC (SPNFeP) formed by nano-precipitation through sonodynamic semiconductor polymer, ferroptosis inducers, and newly synthesized proteolysis-targeting chimera (PROTAC) molecules, can deliver activated PROTAC molecules to tumor sites to degrade nicotinamide phosphoribosyltransferase (NAMPT) thereby inhibiting the infiltration of tumor MDSCs, promoting anti-tumor immunity and suppressing tumor growth (136). Moreover, coating T cell membranes that highly express PD-1 on the surface of PLGA@MnO_2_ nanoparticles loaded with the STING activator 2’3’-cGAMP (PSMP), this nanodrug effectively disrupts the pre-metastatic niche (PMN) by blocking high PD-L1 expression on MDSCs and activating the STING signaling pathway thereby inhibiting metastasis (137). In summary, nanoparticles assembled from dendritic macromolecules or liposomes, as well as nanoparticles composed of various other materials, can regulate antitumor immunity by reducing the number of MDSCs or inhibiting the immunosuppressive function of MDSCs, thereby exerting antitumor effects.

## 7 Nanoparticles regulate T cells

**Figure 5:**
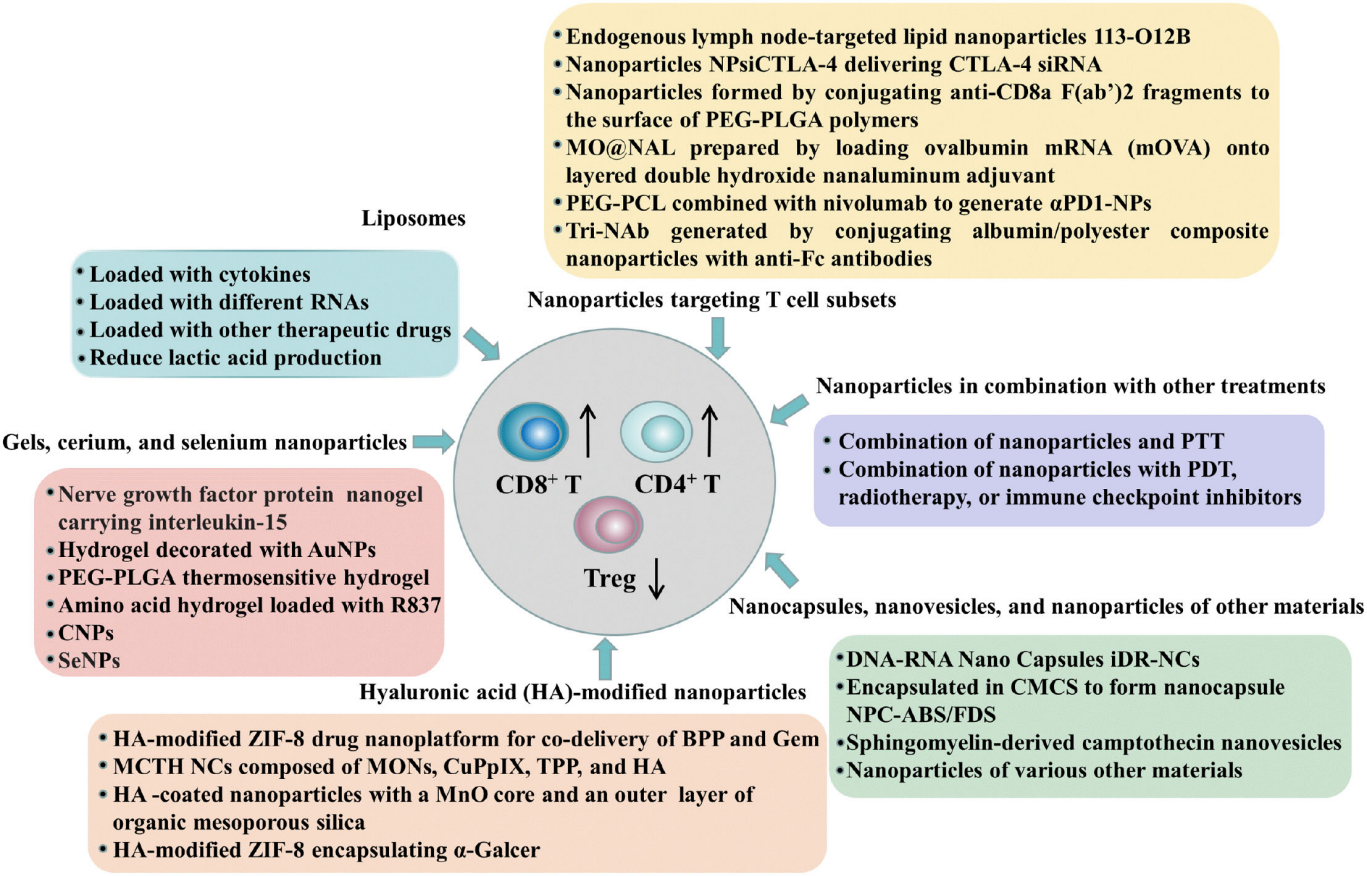
Nanoparticles that regulate antitumor immunity by affecting T cell subsets or their activity: including nanoparticles composed of liposomes, gels, cerium, and selenium; hyaluronic acid-modified nanoparticles; nanocapsules; nanovesicles; and nanoparticles made from other materials, as well as nanoparticles combined with other therapies (in combination with photothermal therapy, photodynamic therapy, radiotherapy, or immune checkpoint inhibitors), and these nanoparticles can exert anti-tumor immune effects by promoting the proliferation or activity of CD8^+^ T cells and reducing the number of regulatory T cells.

T cells are part of the adaptive immune system and cellular components are engulfed after tumor cell lysis and expressed on antigen-presenting cells (APCs) and further exposed to mature lymphocytes thereby resulting in tumor suppression (138). Different subsets of T cells determine the prognosis of tumors, among which the T cell subsets that are conducive to tumor prognosis and survival mainly include: high levels of CD8^+^ and CD4^+^ T cells, high helper T cell (Th)1 to Th2 ratio, and high CD45RO memory T cells (139, 140), while the T cell subsets associated with tumor progression and poor prognosis are predominantly Tregs (141, 142). A variety of nanoparticles have been shown to modulate anti-tumor immunity by affecting T cell subsets or their activity, including nanoparticles composed of liposomes, gels, CNPs, SeNPs, nanoparticles modified with hyaluronic acid, nanocapsules, nanovesicles, as well as nanoparticles used in combination with other therapies (**Figure 5**), and we will introduce in detail the nanoparticles that affect T cells in the following sections.

### 7.1 Liposomes

Liposomes loaded with cytokines, different RNAs, and carrying other therapeutic drugs have been proven to exert anti-tumor effects by inducing the proliferation of ACT T cells, increasing CD8^+^ T cells infiltration or their activity, or reducing the number of Tregs, such as pegylated liposomes using an F(ab’)2 fragment of a unique cell surface antigen (Thy1.1) on ACT cells or an engineered IL-2 molecule on an Fc framework as targeting ligands to treat tumor bearing mouse can not only target ACT cells but also induce ACT T cells proliferation and stimulate ACT T cells to improve the efficacy of ACT (143). In addition, anchoring IL-2 and anti-CD137 on the surface of liposomes not only allows these immune agonists to rapidly accumulate in tumors, but also induces a large proliferation of CD8^+^ T cells and increases the intratumoral CD8/Treg ratio, thereby producing strong anti-tumor activity (144). In addition to liposomes loaded with cytokines, lipid nanoparticle (LNP) carriers of different RNAs can also affect T cell subsets. For example, the ionizable lipid material DAL4-LNP containing diaminic groups of different head groups (DAL) can efficiently deliver different mRNAs to tumor cells, and intratumoral injection of DAL-LNPs loaded with IL-12 and IL-27 mRNA can induce CD8^+^ T cell infiltration into tumors and significantly inhibit tumor growth compared to IL-27 and GM-CSF mRNA monotherapy (145). In addition, lipid nanoparticles encoding the tumor-associated antigens gp100 and TRP2 mRNA can also induce CD8^+^ T cell activation thereby shrinking tumors and prolonging the overall survival of treated mice (146). At the same time, indoleamine 2,3-dioxygenase-1 (IDO1) siRNA was delivered to tumor-draining lymph nodes (TDLNs) and tumor tissues via cationic lipid-assisted nanoparticles (CLAN), the results showed that co-administration of oxaliplatin and CLAN-siIDO1 could achieve a synergistic anti-tumor effect by increasing the number of infiltrating T lymphocytes and reducing the number of Tregs (147). Moreover, encapsulating mRNA encoding tumor-specific toxic proteins neutrophil elastase (ELANE) or porcine pancreatic elastase (PPE) in lipid nanoparticles to form ELANE or PPE mRNA-LNP can both induce CD8^+^ T cell infiltration and significantly inhibit tumor growth (148). In addition to the previously mentioned liposomes loaded with cytokines and various RNAs, liposomes carrying other therapeutic drugs can also exert anti-tumor immune effects by increasing CD8^+^ T cell infiltration or their activity or reducing Tregs, such as lipid nanoparticles α-melitoxin-NPs loaded with melitoxin can induce systemic anti-tumor responses by promoting the release of whole tumor antigens *in situ*, and which can significantly enhance LN accumulation and APCs activation compared with free melitoxin thereby increasing antigen-specific CD8^+^ T cellular response and significantly inhibiting primary and distant tumor growth (149). In addition, modulating tumor acidity can also inhibit tumor growth by activating tumor-infiltrating immune cells, such as the use of *in vivo* optimized vesicle cationic lipid-assisted nanoparticles to mediate systemic knockout of lactate dehydrogenase A (LDHA) in tumor cells can lead to pyruvate metabolism reprogramming, decreased lactate production, and neutralization of tumor pH, thereby increasing CD8^+^ T cell infiltration and reducing the number of immunosuppressive T cells and significantly inhibiting tumor growth (150). In conclusion, liposomes loaded with cytokines, various RNAs, and multiple other therapeutic drugs all can exert anti-tumor immunity by inducing the proliferation of ACT T cells, increasing CD8^+^ T cell infiltration or their activity, or reducing the number of Tregs.

### 7.2 Gels, cerium, and selenium nanoparticles

In addition to liposomal nanoparticles, functionalized gels, cerium nanoparticles, and selenium nanoparticles can exert anti-tumor effects by affecting T cell subsets or their activity. For example, using nerve growth factor protein nanogels (NGs) carrying IL-15 superagonist complexes to treat tumors can selectively expand T cells within the tumor, thereby significantly increasing the tumor clearance rate compared to free cytokines (151). In addition, polymer polyethylene glycol (PEG) hydrogels are crosslinked with two fibronectin-derived peptides and surface-decorated with a quasi-hexagonal array of gold nanoparticles (AuNPs), these particles show higher T cell activation and enhanced T cell proliferation compared to non-functionalized PEG hydrogels (152). Moreover, PEGylated PLGA (PEG-PLGA) thermosensitive hydrogels nsTPPgels with non-shrinking properties loaded with bispecific anti-prostate-specific antigen (PSMA) Fab/anti-CD3 scFv T cell engagers (BiPTE), can promote CTLs migration to tumor sites and initiate T cells immunotherapy through the PSMA-targeting arm on BiPTE thereby enhancing the therapeutic effect against solid tumors (153). At the same time, biodegradable amino acid hydrogels R837@Gel, constructed using fluoromethyl carbamate phenylalanine (Fmoc-Phe) and diphenylalanine (Phe2) substrates and loaded with the Toll-like receptor agonist R837, can release R837 after subcutaneous injection, promoting the proliferation of CD4^+^ T and CD8^+^ T cells and significantly enhancing PD-1 efficacy (154). In addition to the gel, activated antigen-specific (P14) and non-specific CD8^+^ T cells treated with cerium oxide nanoparticles (CNPs) can not only produce more cytokines such as IL-2 and TNF-α but also release more effector molecules such as granzyme B and perforin, and can increase the cytotoxic activity of CTLs (155). Moreover, cerium dioxide (CeO_2_) nanoparticles can significantly reduce the CD4+ PD-1^high^ T cells driving malignancy and activate CD8^+^ T cells to restore their powerful anti-tumor function, significantly improving survival rates (156). Not only that, SeNPs can also enhance anti-tumor immune responses by exerting immunomodulatory effects, for example, SeNPs pretreated γδ T cells can significantly up-regulate the expression of cytotoxicity-related molecules such as NKG2D, CD16 and IFN-γ compared with γδ T cells treated alone, while down-regulating the PD-1 expression of γδ T cells and significantly enhancing the antitumor activity of Vγ9Vδ2 T cells (Vγ9Vδ2 T cells are a subset of peripheral γδ T cells that have been shown to have good antitumor activity), with stronger tumor killing and tumor growth inhibition effects (157). Meanwhile, selenium nanoparticles carrying mouse double minute 2 (MDM2) targeted peptide inhibitor MI (Se@MI) can not only restore p53 function by disrupting the MDM2-p53 interaction, but also increase CD8^+^ T cells infiltration and cytotoxic function, significantly inhibiting tumor growth (158). Not only that, the self-assembled SA formed by peptide a and nano-selenium can also increase CD8^+^ T cells and enhance their function, reduce Tregs thereby reshaping the TME, showing superior anti-tumor performance (159). In summary, functionalized gels, cerium nanoparticles, and selenium nanoparticles can all exert anti-tumor effects by affecting T cell subsets or their activity.

### 7.3 Hyaluronic acid-modified nanoparticles

Hyaluronic acid-modified nanoparticles can also exert antitumor effects by increasing CD8^+^ T cell infiltration or activating T cell-mediated immunity. For example, the hyaluronic acid -modified zeolitic imidazolate framework-8 drug nanoplatform HA/ZIF-8@BPP/Gem which co-delivers phosphorophenylpiperidine and gemcitabine can stimulate the secretion of immune-related cytokines and activate T cell-mediated immunity thereby improving tumor treatment efficacy (160). In addition, MCTH NCs composed of disulfide (S-S) doped mesoporous organosilica (MONs), copper-modified porphyrin (CuPpIX), mitochondria-targeted triphenylphosphine (TPP), and CD44-targeted hyaluronic acid, can efficiently accumulate at tumor sites, significantly enhance CD8^+^ T cell infiltration into tumors thereby achieving potent antitumor acoustic immunotherapy (161). In addition, the mitochondrial glycolysis inhibitor lonidamine (LT) is encapsulated in a nanomedicine LT@MnO@MON-HA (LMMH) with a manganese oxide (MnO) core and an organic mesoporous silica (MON) shell and coated with hyaluronic acid, which acts as an agonist of the cGAS-STING pathway to stimulate cytokine release and activate effector T cells after reaching the tumor site thereby triggering a systemic immune response (162). Hyaluronic acid-modified metal-organic frameworks (zeolitic imidazolate framework-8, ZIF-8) encapsulating α-galactosylceramide α-Galcer/DOX@ZIF-8@HA gradually disassemble in the TME to release α-Galcer, activating NKT cells and initiating an anti-tumor immune cascade response (163). In summary, hyaluronic acid-modified nanoparticles can also exert anti-tumor effects by affecting T cell subsets or their activity.

### 7.4 Nanocapsules, nanovesicles, and nanoparticles of other materials

In addition to the aforementioned nanoparticles, nanocapsules, nanovesicles, and nanoparticles composed of various other materials can also exert anti-tumor effects by increasing CD8^+^ T cells or their activity, or by reducing Tregs. For example, a type of self-assembled intertwined DNA-RNA nanocapsule (iDR-NCs) can efficiently deliver synergistic DNA CpG and short hairpin RNA (shRNA) adjuvants as well as tumor-specific peptide neoantigens to antigen-presenting cells (APCs) in lymph nodes, which induced neoantigen-specific peripheral CD8^+^ T cell frequency is 8 times higher than that of CpG alone, inducing T cell memory and significantly inhibiting tumor progression (164). In addition, baicalein (BAE) and DOX were separately loaded into cationic solid lipid nanoparticles to form BAE-SLN and DOX-SLN, where BAE-SLN was modified with aminoethyl aniline amide (AEAA) to form AEAA-BAE-SLN (ABS) and DOX-SLN was modified with folic acid (FA) to form FA-DOX-SLN (FDS), and both were further encapsulated in carboxymethyl chitosan (CMCS) to form the nanocapsule NPC-ABS/FDS which can enhance CD8^+^ T and CD4^+^ T cells infiltration, reduce Tregs, and significantly inhibit tumor growth (165). Furthermore, sphingomyelin-derived camptothecin nanovesicles (camptothecin) can not only induce a potent granzyme-B and perforin-mediated CTLs response but also enhance the blocking effect of PD-L1/PD-1 thereby eliminating tumors (166). Moreover, nanoparticles composed of various other materials have also been shown to exert anti-tumor effects by modulating T cell subsets or their activity. For example, chitosan (CS) can exhibit adjuvant effects when used as a vehicle to drive an effective immune response, such as repeated inhalation of CS with anti-PD-L1 assembly to form the CS/aPD-L1 complex can activate the immune system by promoting the infiltration of different immune cells especially CD8^+^ T cells around tumor lesions and ultimately prolong the survival of mouse (167). Moreover, DOX/aNLG919-loaded CaCO_3_ nanoparticles (DNCaNPs) obtained by combining the ICD inducer DOX and the IDO1 inhibitor alkylated NLG919 can induce ICD in cancer cells and can induce effective anti-tumor immune responses by inhibiting IDO1 to limit the production of immunosuppressive kynurenine thereby leading to increased tumor cytotoxic CD8^+^ T cell infiltration and immunosuppressive Tregs depletion, and the combination of chemotherapy and immunotherapy with this nanoparticles can effectively inhibit tumor growth (168). At the same time, tumor-targeted lipid-terminal matrix calcium phosphate (TT-LDCP) nanoparticles TT-LDCP NPs constructed by thymine functionalized dendritic macromolecules can also increase the tumor infiltration and activation of CD8^+^ T cells by delivering anti-immune checkpoint ligand PD-L1 and immunostimulatory IL-2-encoded plasmid DNA siRNAs to tumors, thereby improving the efficacy of cancer vaccine immunotherapy and inhibiting tumor progression (169). In addition, cancer cell membrane-camouflaged gelatin nanoparticles (CSG@B16F10) can co-deliver the oxygen-producing catalase (CAT) and CD73siRNA to enhance tumor oxygen and alleviate CD73-adenosine (CD73-ADO)-mediated T cell immunosuppression, promote T-cell-specific immunity and lead to CTLs enhancement and Tregs reduction thereby enhancing immune efficacy (170). Moreover, 3-(2-nitrophenyl) propionate-piperidinol nanoparticles (NPPA-PTX NPs) can recruit infiltrating CD3, CD4, and CD8 T cells, secrete IFN-γ and TNF-α, reactivate antitumor immunity, and can further stimulate antitumor immune responses and enhance antitumor activity when combined with aPD-L1 (171). In summary, nanoparticles composed of various materials can exert anti-tumor effects by increasing the number or activity of T lymphocytes or reducing Tregs, such as liposomes, gels, CNPs, SeNPs, hyaluronic acid-modified nanoparticles, nanocapsules and nanovesicles.

In addition to the aforementioned nanoparticles, there are some nanoparticles that can regulate T cells by directly targeting T cell subsets, thereby exerting antitumor immune effects. For example, an endogenous lymph node-targeted lipid nanoparticle 113-O12B shows higher and more specific expression in the lymph node (LN), and LN-directed delivery of mRNA enhances the CD8^+^ T cell response to the full-length ovalbumin model antigen and improves antitumor efficacy (172). In addition, the delivered cytotoxic T-lymphocyte associated antigen-4 (CTLA-4) siRNA (NPsiCTLA-4) can deliver CTLA-4 siRNA to CD4 (+) and CD8 (+) T cell subsets at the tumor site, significantly increasing CD8 (+) T cells and reducing the proportion of Tregs, while also enhancing the activation of tumor-infiltrating T cells and antitumor immune responses (173). Moreover, nanoparticles formed by conjugating anti-CD8a F(ab’)2 fragments to the surface of maleimide-functionalized PEG-PLGA polymers can target CD8^+^ T cells in the blood, lymphoid tissues, and tumors of mice, increase the proportion of tumor-infiltrating CD8^+^ T cells and make tumors more sensitive to anti-PD-1 therapy (174). A bifunctional immunomodulator MO@NAL prepared by loading ovalbumin mRNA onto lysozyme-coated layered double hydroxide nanoalum adjuvants, can be internalized by tumor cells and efficiently mark the tumor cell surface with the carried mOVA, turning the tumor cells into targets that recruit and direct antigen-specific cytotoxic T cells thereby destroying the tumor cells (175). Meanwhile, αPD1-NPs are generated by combining polyethylene glycol-poly(ϵ-caprolactone) (PEG-PCL) nanoparticles with nivolumab, which can target PD1-overexpressing tumor-infiltrating lymphocytes and reverse the exhaustion of PD1+CD8+ TILs when used in combination with the epigenetic drug 5-aza-2’-deoxycytidine (DAC), improve T cell responses and enhance the efficacy of immune checkpoint blockade therapy (176). In addition, three monoclonal antibodies (mAbs) were immobilized to generate a trispecific nanobody Tri-Nab by using optimized albumin/polyester composite nanoparticles conjugated with anti-Fc antibodies, which can simultaneously target PDL1, 4-1BB, and NKG2A (or TIGIT), and can effectively bind NK cells and CD8^+^ T cells, inducing their activation and proliferation thereby achieving efficient tumor killing (177). In summary, various nanoparticles have been shown to regulate T cells by directly targeting T cell subsets, these nanoparticles can not only enhance immune responses but also reduce systemic side effects.

### 7.5 Nanoparticles in combination with other treatments

Nanoparticles can also induce or enhance anti-tumor T cells immunity in combination with other treatments, such as photothermal therapy (PTT), photodynamic therapy (PDT), radiotherapy, and immune checkpoint inhibitors (ICIs), and we will detail the strategy of nanoparticles in combination with other therapies to modulate T cells in TME thereby inhibiting tumors in the following content.

#### 7.5.1 Combination of nanoparticles and PTT

PTT thermal ablation of local tumors is a promising minimally invasive method which can be used in combination with nanoparticles to exert anti-tumor immunity by modulating T cells in TME or enhancing the ability of nanoparticles to regulate T cells. For example, the magnetic nanoparticle Fe_3_O_4_-ICG@IRM formed by fusing the membrane of mouse-derived ID8 ovarian cancer cells with red blood cell (RBC) membrane to form a hybrid biomimetic coating (IRM) and further loading ICG for the treatment of cancer can not only release tumor antigens by photothermal-induced tumor necrosis, but also enhance the anti-tumor immune of primary and metastatic tumors by activating CD8^+^ T cells and reducing regulatory Foxp3^+^ T cells (178). In addition, systemic administration of PTT mediated iron oxide nanoparticles (IONP) can also improve the anti-tumor efficacy of checkpoint blockade-based cancer immunotherapy by depleting immunosuppressive Tregs in tumor tissues thereby inhibiting tumor growth, and the combination therapy based on IORP-mediated sequential PTT can generate memory tumor antigen-specific CD8^+^ T cells thereby preventing tumor recurrence (179). At the same time, αPDL1 nanoparticle treatment combined with the activation of the photosensitizer indocyanine green triggered by NIR laser irradiation can induce the production of reactive oxygen species thereby promoting CTLs infiltration into tumors and sensitizing tumors to PD-L1 blockade therapy and effectively inhibiting tumor growth and metastasis (180). In summary, the combination of nanoparticles and PTT can exert an anti-tumor effect by activating CD8^+^ T cells in the TME, promoting CD8^+^ T cell infiltration, or reducing Tregs. In addition, the combined application of nanoparticles and PTT can enhance the former’s ability to regulate T cells thereby exerting a stronger anti-tumor effect. For example, bovine serum albumin (BSA) nanoparticles loaded with aggregation-induced emission (AIE) photosensitizers (BSA/TPA-Erdn) can enhance ICD to activate T cells and reverse T cell senescence, thereby restoring the antitumor immune microenvironment (181). Moreover, smart semiconducting polymer nanoimmunomodulators (SPNIs) are formed by the self-assembly of a NIR absorbing semiconductor polymer and an amphiphilic polymer conjugated to a TLR7 agonist by an acid-base linker can release immunogenic factors and TLR7 agonists in response to acidic TME, this localized immune activation can enhance systemic anti-tumor immune responses, thereby increasing cytotoxic CD8^+^T cells infiltration and inhibiting tumor growth and metastasis (182). At the same time, the poly(lactic-co-glycolic acid)-indocyanine green (PLGA-ICG) component (PI) implanted eukaryotic-prokaryotic vesicle (EPV) nanoplatform PI@EPV can significantly enhance CTL-induced tumor-specific immunity and show synergistic anti-tumor effects after combined laser irradiation (808 nm) and further enhances CD8^+^ CTLs infiltration in the combination treatment group compared with the unirradiated treatment thereby resulting in a stronger tumor inhibitory effect (183). Not only that, a novel immunogold nanoparticle AuNP@B16F10 developed through intracellular generation and excretion by tumor cells is further internalized by DCs and secreted as DC-derived vesicles, in which the introduction of DC2.4 allows it to enhance T cell proliferation through indirect mediation of APC and direct interaction with T cells, and the infiltration of CD4^+^ T cells and CD8^+^ T cells increased after AuNP@DCB16F10 combined with NIR, which further inhibited or even eliminated the primary tumor, metastatic tumor and tumor recurrence, and ultimately improved the overall survival rate of mouse (184). In conclusion, the combination of PTT and nanoparticles can exert anti-tumor immunity by modulating T cells in TME or enhancing the ability of nanoparticles to regulate T cells.

#### 7.5.2 Combination of nanoparticles with PDT, radiotherapy, or ICIs

In addition to exerting anti-tumor effects in combination with PTT to regulate T cells in TME, nanoparticles can also be combined with PDT, PDT is a non-invasive, effective treatment for local tumors, and can be used to treat local tumors that are palpable by the light source. Studies have demonstrated that nanoparticle-mediated PDT can exert anti-tumor efficacy by activating the innate immune system and adaptive immune system in the TME. For example, zinc pyrophosphate (ZnP) nanoparticles ZnP@pyro loaded with photosensitizer pyrolipid combined with anti-PD-L1 therapy under light can induce CD8^+^ T cell infiltration into distant tumor tissue and increase the activity of CD8^+^ T cells, while also reducing Tregs to eradicate the primary tumor and inhibit distant tumor (185). Moreover, mesoporous silica nanoparticles (bMSNs) carrying multiple neoantigen peptides, CpG oligonucleotide adjuvants, and photosensitizer chlorine e6 can not only efficiently accumulate in tumors but also recruit DCs to the tumor site of PDT treatment by combining laser irradiation with PDT and induce neoantigen-specific CTLs to exert strong anti-tumor immunity against locally treated tumors as well as distant untreated tumors (186). At the same time, phase change core-shell nanoparticles OIX_NPs carrying oxygen in the nucleus and photosensitizer ICG/Oxaliplatin (OXP) in the shell can significantly enhance anti-tumor immunity and inhibit the growth of primary and distant tumors by increasing the intratumoral infiltration of CTLs in mouse tumor models (187). Not only that, the IDoi@TBC-Hf nanoparticles formed by loading the IDO inhibitor (IDoi) indoleamine 2,3-dioxygenase into TBC-Hf (a new nMOF) using the highly porous structure of the nanometal-organic framework (nMOF) can reverse the inhibitory tumor microenvironment by releasing IDoi after local injection, and combined with PDT can induce ICD and further stimulate the immune system, leading to infiltration of CD4^+^ T and CD8^+^ T cells in the TME and effectively controlling both local and distant tumors (188). Nanoparticles combined with radiotherapy or ICIs can also further enhance T cells recruitment in addition to the combination of PTT and PDT, such as the core-shell nanoparticle PLGA-R837@Cat prepared by encapsulating water-soluble catalase (Cat) and TLR7 agonist imiquimod (R837) in the PLGA shell can alleviate tumor hypoxia and modulate the immunosuppressive tumor microenvironment and not only recruit CD8^+^ CTLs and helper T cells into tumors and reduce the percentage of Tregs but also lead to higher CTLs infiltration in secondary tumors when combined with radiotherapy and CTLA-4 checkpoint blockade (αCTLA4) to treat mouse tumors thereby inducing a strong anti-tumor immune response and effectively inhibiting tumor metastasis (189). In addition, polymeric nanovesicles Gem/Sul-NP which simultaneously encapsulate and deliver gemcitabine and the prolyl isomerase Pin1 inhibitor sulfopin, can effectively remodel the immunosuppressive TME and significantly increases the infiltration of CD8 IFNγ T cells when combined with anti-PD-1 therapy (190). In conclusion, nanoparticles combined with PTT, PDT, radiotherapy, and ICIs can also exert anti-tumor immunity by modulating T cells in TME.

## 8 Nanoparticles simultaneously regulate various immune cells

In addition to the nanoparticles that regulate TAMs, NK cells, DCs, TANs, MDSCs, and T cells, many nanoparticles can also improve anti-tumor immunity by simultaneously modulating multiple immune cells, such as affecting NK cells and T cells, affecting TAMs and T cells, affecting DCs and T cells, and affecting multiple immune cells simultaneously.

### 8.1 Nanoparticles affecting NK cells and T cells

A variety of nanoparticles have been shown to be able to simultaneously regulate NK cells and T cells to improve anti-tumor immunity, such as the zinc ions released by the nanoclusters of zinc-sulfur@bovine serum albumin in the acidic tumor microenvironment by the self-assembly method can significantly enhance the signal of cyclic guanosine-adenosine monophosphate synthase/interferon gene stimulator (cGAS/STING) thereby promoting the infiltration of CD8^+^ T cells and the cross-presentation of NK cells at the tumor site, and improving the effectiveness of tumor immunotherapy (191). In addition, lipid nanoparticles delivering 5T4 and CD70 mRNA can also enhance CD8^+^ T cells and NK cells activity, showing better tumor suppression effects and prolonged survival (192). Moreover, nanoparticle crystals Ali-Rux composed of the Aurora kinase inhibitor alisertib (Ali) and the JAK2 inhibitor ruxolitinib (Rux), can remodel the TME by increasing the recruitment and activation of CD8^+^ T cells and NK cells while reducing MDSCs, and can trigger a lasting anti-tumor immune response when combined with PD-L1 blockade (193). At the same time, chemotherapy-induced tumor RNA nanoparticles C-RNA-NPs can also improve cancer immunotherapy, and which can not only be captured by NK cells and stimulate NK cells maturation but also promote the infiltration of intratumor T cells and increase the ratio of CD8^+^ T cells to Tregs thereby improving the efficacy of immune checkpoint blockade (ICB) therapy and improving cancer immunotherapy (194). In summary, some nanoparticles can simultaneously regulate NK cells and T cells in TME to exert antitumor effects.

### 8.2 Nanoparticles affecting TAMs and T cells

There are also some nanoparticles that can simultaneously regulate TAMs and T cells to exert anti-tumor immunity in addition to the nanoparticles that regulate both NK cells and T cells, such as the DNA nanodevice E64-DNA targeting the lysosomes of TAMs in mouse can not only reprogram TAMs to improve their ability to cross-present antigens by inhibiting cysteine proteases that are specifically present within TAMs lysosomes but also inhibit tumor growth by affecting CD8^+^ T cells (195). In addition, the multivalent bispecific nanobioconjugated conjugated conjugator mBiNE created by colloidal nanoparticles as substrates can stimulate human epidermal growth factor receptor 2 (HER2)-targeted phagocytosis by simultaneously targeting HER2 and calreticulin-mediated prophagocytosis expressed by cancer cells and generate durable anti-tumor immune responses against HER2-expressing tumors, such as promoting macrophages recruitment and enhancing the phagocytosis of cancer cells by macrophages, promoting higher T cell infiltration and activation, thereby further eliminating cancer cells and improving antitumor efficacy (196). At the same time, a sequentially targeted sonodynamic nanovaccine Stars NV not only can increase the local M1/M2 TAM ratio and tumor-infiltrating lymphocytes while decreasing Tregs, but also can enhance systemic immunity, increase peripheral CD4^+^ T and CD8^+^ T cells and ultimately extend the survival of mice (197). In addition, nanoparticles based on polysaccharides (chitosan or hyaluronic acid) can also simultaneously affect the number or activity of TAMs and CD8^+^ T cells to exert antitumor effects. For example, a nanogel PMNG carrying the chemotherapy drug doxorubicin was prepared by crosslinking carboxymethyl chitosan (CMCS)-derived multiple metformin (PolyMetCMCS) with cystamine, and then coated with hyaluronic acid on its surface to form the nanogel D@HPMNG, which can effectively remodel the TME by reprogramming the phenotype of TAMs and recruiting intratumoral CD8^+^ T cells thereby significantly inhibiting tumor growth (198). Not only that, the polysaccharide PSM001 isolated from the kernels of Mangifera indica Kottukonam variety and the galactoxyloglucan (PST001) isolated from Tamarindus indica seeds, both have immunostimulatory potential and can be used as immunomodulators for tumor treatment (199). For example, copper nanoparticles CuNP@PST formed from PST001 extracted from the embryo of Tamarindus indica seeds and CuSO_4_·5H_2_O, can upregulate the M1 marker iNOS when co-cultured with macrophages *in vitro* and can stimulate humoral and cellular immunity after administration *in vivo*, leading to an increase in CD4^+^ T and CD8^+^ T cells, enhanced expression of cytokines IL-2, IFN-γ, IL-4, IL-12, IL-6, Eotaxin, MIP-2, RANTES, TNF-α, and thrombopoietin, thereby reducing tumor burden and significantly prolonging the survival of tumor-bearing mice (200). In other words, some nanoparticles can simultaneously regulate TAMs and T cells in TME to inhibit tumor growth.

### 8.3 Nanoparticles affecting DCs and T cells

Nanoparticles composed of polysaccharides or other materials have been shown to exert antitumor immunity by simultaneously affecting DCs and T cells. For example, loading the photosensitizer IR780 and the immunotherapeutic drug imiquimod (IMQ) into temperature- and pH-responsive chitosan-based nanoparticles to form EpCAM-CS-co-PNVCL@IR780/IMQ chitosan nanoparticles can effectively induce immunogenic ferroptosis of cancer cells, promote DCs maturation and subsequently activate CTLs to exert antitumor immunity (201). In addition, encapsulating mRNA encoding tumor neoantigens into mannose-modified chitosan ethosomes (EthsMC) to obtain a multivalent mRNA vaccine (MmRV) can effectively induce DCs maturation, promote CD4^+^ T and CD8^+^ T cells infiltration in tumor tissues and inhibit tumor growth (202). Moreover, the multifunctional sodium alginate rapidly cross-linked to form a hydrogel in the presence of physiological concentrations of Ca^2+^, which can slowly and continuously release loaded hyaluronidase, DOX, and micellar IP-NPs, thereby effectively promoting the maturation of DCs and activating T lymphocytes, ultimately generating long-term immune memory (203). At the same time, the multifunctional nano-porphyrin material (P18-APBA-HA) constructed using photosensitizer-purpurin 18 (P18), hyaluronic acid, and 4-(aminomethyl)phenylboronic acid (APBA) can be inserted into the phospholipid membrane and loaded with epacadostat (EPA) to prepare a dual drug delivery system LipEPAP18-APBA-HA, which can also induce anti-tumor immunity by promoting DCs maturation, activating CD8^+^ T cells and inhibiting Tregs thereby suppressing tumor growth and metastasis (204). In addition, multifunctional nanoparticles HA/AIPH@ZIF-8 (HAZ) containing hyaluronic acid where dsDNA and zeolitic imidazolate framework (ZIF-8)-derived zinc ions can promote the STING pathway, and mediate DCs maturation and CTLs infiltration through ICD thereby enhancing tumor immunotherapy (205). Not only that, biodegradable Mn-doped mesoporous silica (MM) nanoparticles with metal-organic framework and hyaluronic acid-modified red blood cell membrane camouflage, loaded with cisplatin and STING agonist SR-717, can trigger cGAS-STING activation, enhance DCs maturation and CTLs infiltration, achieving efficient tumor regression (206). In addition to polysaccharide-based nanoparticles, pH-responsive loaded micelles OPDEA-PGED-5HA@Cur@Rg3 (PPH@CR) can also promote DCs maturation by inducing ICD in tumor cells, thereby increasing the infiltration of CD4^+^ T and CD8^+^ T cells in tumor tissues and spleen tissues to exert anti-tumor effects (207). In addition, the nanoprotein hydrolytic targeted chimera (PROTAC) formulation ARV@PEG-ICG composed of indocyanine green-functionalized polyethylene glycol (PEG-ICG) phototherapeutic agent and BRD4 degrader ARV-825, rapidly decomposes under activation in the acidic tumor microenvironment thereby inducing ICD, promoting DCs maturation and T cells activation and inhibiting tumor growth (208). Moreover, PLGA NPs nanoparticles are formed by PLGA nanoparticles (NPs) that encapsulate heparanase CD4^+^ and CD8^+^ T cell epitopes alone or in combination with TLR 3 and 7 ligands as model antigens and then use DEC-205 antibodies to target the ligand to DCs surface molecules can not only target DCs more effectively and be internalized by DCs but also promote T cells proliferation, showing higher tumor killing efficacy *in vitro* and *in vivo (*209) In summary, nanoparticles composed of polysaccharides or other materials can exert anti-tumor activity by simultaneously regulating DCs and T cells.

### 8.4 Nanoparticles that affect a variety of immune cells

Nanoparticles composed of polysaccharides, gels, polymers, and many other materials have been shown to exert antitumor activity by simultaneously affecting multiple immune cells. For example, chitosan oligosaccharide (COS)-dendritic polycarbonate (DPC) nanoparticles (CD NPs) can significantly activate TBK1/IRF3 phosphorylation, enhance STING signaling in macrophages and tumor cells, increase M1 TAMs and promote DCs maturation, reduce MDSCs and facilitate CD8^+^ T cells infiltration thereby reshaping the tumor microenvironment into a pro-inflammatory state (210). A light-controlled polysaccharide nano-immunomodulator Dex-NB-CPT can rapidly release the ICD-inducing agent camptothecin under light irradiation thereby activating DCs and cytotoxic T cells and further promoting macrophage M1 polarization, significantly inhibiting tumor growth (211). In addition, the biodegradable sodium alginate hydrogel S1P-αPDL1@Ge which delivers sphingosine-1-phosphate (S1P) and anti-PD-L1 (αPDL1), can significantly enhance the infiltration of DCs, M1 macrophages, CD4^+^ T cells and CD8^+^ T cells, effectively inhibit tumor growth and reduce local tumor recurrence (212). Moreover, the complex Gel/(REG NG/LY) is formed by TGF-β inhibitor LY3200882 (LY) encapsulated in ROS-responsive nanogels and homogeneously dispersed with regorafenib (REG) in a thermosensitive hydrogel can not only reduce the recruitment of TAMs and MDSCs and promote macrophages polarization from M2 to M1, but also increase the infiltration of CD8^+^ T cells into tumors thereby effectively inhibiting tumor growth and metastasis (213). Meanwhile, the novel thermosensitive hydrogel SCC15 PLHCu@Gel loaded with disulfiram derivative CPD12C15 (SCC15) and tumor-targeted Cu²^+^ (PLHCu), can inhibit tumors by enhancing tumor-infiltrating cytotoxic CD8^+^ T cells, promoting DCs maturation, and suppressing Tregs and MDSCs (214). Furthermore, polymeric nanoparticles can also exert anti-tumor immunity by affecting multiple immune cells simultaneously. For example, the multifunctional nano-epigenetic inhibitor CREDIT composed of polymeric polylactic acid-glycolic acid (PLGA) grafted with histidine (PLGA-his) loaded with the epigenetic inhibitor TMP195, modified with red blood cell membranes and conjugated with the TAM-targeting peptide CRV, can reprogram M2-type TAMs into the M1 phenotype, reduce MDSCs, increase the number of T cells in tumors and inhibit tumor growth (215). The mRNA delivery of polymer nanoparticles can effectively induce the expression of Phosphatase and Tensin Homolog (PTEN) in cancer cells, based on this, the PTEN mRNA nanoparticles can reverse the immunosuppressive tumor microenvironment by promoting CD8^+^ T cells infiltration into tumor tissues and reducing Tregs and MDSCs, showing highly effective anti-tumor effects when used in combination with PD-1 antibodies (216). Besides, a TME-responsive targeted nanoparticle co-delivering the gene expressing interleukin-12 and the colony-stimulating factor-1 receptor inhibitor PLX3397 can efficiently inhibit tumor growth and metastasis by stimulating the proliferation and activation of T lymphocytes, repolarizing TAMs, reducing MDSCs, promoting DCs maturation, and promoting the secretion of anti-tumor cytokines (217). Not only that, the nanoparticle vaccine R848-GA@TCLs based on sulfite-modified Toll-like receptor 7/8 (TLR7/8) agonist R848, can promote the production of pro-inflammatory cytokines, activate DCs, and significantly increase the number of effector T cells, NK cells, and M1 TAMs, markedly inhibiting tumor growth and metastasis in various tumor models (218). At the same time, the nanoparticle ppp-dsRNA that delivers Bcl-2-specific short interfering double-stranded RNA (dsRNA) through lipid calcium phosphate nanoparticles can also strongly induce the level of pro-inflammatory Th1 cytokines, increase the proportion of M1 TAMs and CD8^+^ T cells and reduce the levels of immunosuppressive B-regulating cells and plasma cells in TME thereby significantly inhibiting tumor growth (219). In summary, nanoparticles can not only exert antitumor activity by regulating TAMs, NK cells, DCs, TANs, MDSCs, and T cells in the TME, but also simultaneously regulate two or more immune cells to exert antitumor immunity, such as simultaneously regulating NK cells and T cells, regulating TAMs and T cells, regulating DCs and T cells, as well as nanoparticles that simultaneously regulate multiple immune cells.

In addition, some nanoparticles can regulate various immune cells through directly targeting immune cell subtypes to exert anti-tumor effects. For example, synergistic sonodynamic immunotherapy mediated by a polymer nanosome-lysosome-targeting chimera nano-LYTAC can degrade membrane proteins on M2-type macrophages and produce sonodynamic effects, and can effectively reprograms the tumor immunosuppressive microenvironment by inhibiting the functions of M2 TAMs and Tregs, as well as promoting DCs maturation and tumor infiltration of effector T cells, thereby inhibiting tumor growth and metastasis (220). Moreover, twin-like charge-switchable nanoparticles shMFN1-NPs + DOX-NPs (MIX-NPs) can selectively target TAMs and cancer cells and deliver shMFN1 by inhibiting mitochondrial fusion, which can not only restructure M2 TAMs to the M1 TAMs but also promote DCs maturation and CD8^+^ T cells infiltration and activation and inhibit MDSCs and Tregs, showing strong anti-tumor efficacy (221). At the same time, liposomal nanoparticles LNP-CDN loaded with cyclic dinucleotides (CDN) can not only protect CDN from enzymatic degradation in malignant pleural effusion (MPE) but also specifically target macrophages and DCs, in addition, LNP-CDN alone or in combination with anti-PD-L1 antibody can not only lead to significant up-regulation of M1-related genes in TAMs, promote NK cells maturation and enhance the cytotoxic activity of NK cells, but also significantly increase the number of CD8^+^ T cells and reduce the volume of MPE, inhibit tumor growth, and ultimately significantly prolonging the survival of MPE tumor-bearing mouse (222). At the same time, a dual-targeted nanovaccine (NV) N-M2T-gp100 HBc NV based on hepatitis B core antigen (HBcAg)-derived virus-like particles (VLPs) can simultaneously target SIGNR DCs/TAMs thereby repolarizing M2-type TAMs and enhancing T cells activity, inhibiting the growth of both local and distant tumors (223). In addition, the nanovaccine Si9GM is made of central radial mesoporous silica nanoparticles coated with membranes from bone marrow-derived dendritic cells (BMDCs) expressing antigen peptides, used to deliver αCLEC9A-antigen conjugates and STING agonists (2′3′-cGAMP), which can selectively target cDC1, promote DCs maturation, and lead to upregulation of cytotoxic T cells, reduction of Tregs, and M1/M2 macrophage polarization and can induce strong tumor growth and metastasis inhibition when combined with αPD-1 blockade (224). In short, various nanoparticles have been shown to exert anti-tumor immunity by directly targeting immune cell subtypes and thereby modulating multiple immune cells in the tumor microenvironment.

**Table 1:** Nanoparticles regulating immune cell subtypes, mechanisms, and references.

Regulatory immune cells	Mechanism	Nanoparticles	References
TAMs	Depletion of M2 TAMs	Nanoparticles in response to MMP-2; Nanoparticles loaded with CSF-1R inhibitors or CSF-1R blockers; Nanoparticles loaded with bisphosphonates; Nanoparticles that inhibit lactic acid production; DAS-MMic micellars for delivery of dasatinib.	(15–25)
	Restricting the recruitment and localization of TAMs	siRNA nanoparticles targeting CX3CL1; Cationic nanoparticles CNP-siCCR2 encapsulated with siCCR2; KLAK-MCP-1 micelles blocking the MCP-1/CCR2 axis; Nanoparticle Cu@CuOx loaded with targeting CCR2 and the chemotherapy drug gemcitabine.	(26–29)
	Reprogramming of M2 TAMs to M1 TAMs	Iron-based nanoparticles; Nanoparticles loaded with TLR agonists; Nanoparticles carrying pathway inhibitors or activators (such as PI3Kγ inhibitors, c-MYC inhibitors, NF-κB and IRF5 activators); Other nanoparticles that reprogram TAMs.	(32–69)
NK cells	Nanoparticles that enhance NK cells homing	Iron oxide nanoparticles; Manganese-containing nano composite Bio-MnS; IMM-MS nanoparticles composed of IFN-γ, IONC, and PLGA; PEG-modified dendrimerentrapped gold nanoparticles Au DENPs; Translational SeNPs.	(73–77)
	Nanoparticles that enhance NK cells function	Selenium nanoparticles; Ruthenium nanoparticles; Nanoparticles loaded with RNA effectors; Nanoparticles loaded with certain genes or factors; Other nanoparticles.	(78–96)
DCs	Promoting DCs maturation	Nanodiscs sHDL; Nano vaccines; Polysaccharide-based nanoparticles; RNA-lipid complex; Gold nanoparticles, etc.	(99–110)
TANs	Inhibiting the recruitment and migration of neutrophils	Nanoparticles loaded with doxorubicin LA/DOX-NP; Paclitaxel prodrug nanoparticles mP-NPs-DNase/PTX;	(112–116)
	Changing the polarity of neutrophils	Lipid-based nanoparticle siTGFβ-PLP-Nes; Nanovaccine encapsulating STAT3 siRNA; Cowpea mosaic virus (CPMV) nanoparticles.	
MDSCs	Reduce circulating and tumor-infiltrating MDSCs, alter MDSCs phenotype or inhibit MDSCs functions	Nanocapsules and metal-organic frameworks: Multilayer polymer nanocapsules; Graphene nanocapsules; Lipid nanocapsules loaded with gemcitabine; hyaluronic acid-modified (M+H)@ZIF/HA nanoparticles; HA/ZIF-8@Gem/D-1-MT nanoparticles obtained by loading Gem and D-1-MT into ZIF-8 and then modifying with hyaluronic acid, etc.	(119–123)
		Micelles and polymers: Micelle system PAH/RGX-104@PDM/PTX encapsulating liver X receptor agonists; Polymeric micelles loaded with 6-thioguanine; Low-molecular-weight heparin-tocopheryl succinate nanoparticle LMWH-TOS; Biomimetic NM/PPcDG/D nansystem; Glutathione-responsive polymer R-mPDV/PDV/DOX/sil.	(124–128)
		Dendritic macromolecules, liposomes, and other material nanoparticles: Dendrimer carrying gemcitabine; Liposomes encapsulating PI3Kγ inhibitors; High-density lipoprotein-like nanoparticles HDL; Nanoparticles SPNPs coated with the cell-penetrating peptide iRGD; Nanoparticles FIT loaded with TAD and indocyanine green; Gemcitabine-celecoxib nanoparticles based on GEM-CXB; Multifunctional nano PROTAC (YM@VBM) based on proteasome-targeting chimeras, etc.	(129–136)
T cells	Promote CD4^+^ T cells and CD8^+^ T cells proliferation, increase CD8^+^ T cells infiltration or activity, and reduce the number of Tregs	Liposomes: Loaded with cytokines; Loaded with different RNAs; Loaded with other therapeutic drugs; Reduce lactic acid production.	(143–150)
		Gels, cerium, and selenium nanopartic: Nerve growth factor protein nanogel carrying interleukin-15; Hydrogel decorated with AuNPs; PEG-PLGA thermosensitive hydrogel; Amino acid hydrogel loaded with R837; CNPs; SeNPs, etc.	(151–159)
		Hyaluronic acid (HA)-modified nanoparticles: HA-modified ZIF-8 drug nanoplatform for co-delivery of BPP and Gem; MCTH NCs composed of MONs, CuPpIX, TPP, and HA; HA-coated nanoparticles with a MnO core and an outer layer of organic mesoporous silica; HA-modified ZIF-8 encapsulating α-Galcer.	(160–163)
		Nanocapsules, nanovesicles, and nanoparticles composed of other materials: DNA-RNA nanocapsules iDR-NCs; Nanocapsules NPC-ABS/FDS loaded with BAE and DOX; Sphingomyelin-derived camptothecin nanovesicles; The CS/aPD-L1 complex is formed by assembling anti-PD-L1 with chitosan; CaCO_3_ nanoparticles DNCaNPs; Calcium phosphate nanoparticles TT-LDCP; Cancer cell membrane-coated gelatin nanoparticles CSG@B16F10; 3-(2-nitrophenyl) propionate-piperidinol nanoparticles; Lipid nanoparticles 113-O12B; Nanoparticles loaded with CTLA-4-siRNA; Nanoparticles αPD1-NPs formed by PEG-PCL combined with PD1.	(164–177)
		Nanoparticles used in combination with other therapies: Combination of nanoparticles and PTT: Magnetic nanoparticles Fe_3_O_4_-ICG@IRM; Iron oxide nanoparticles IONP; αPDL1 nanoparticles; Bovine serum albumin nanoparticles loaded with BSA/TPA-Erdn; Polymeric nano-immunomodulator SPNI; Eukaryotic-prokaryotic vesicle nanoplatform PI@EPV; Gold nanoparticles AuNP@B16F10. Combination of nanoparticles with PDT, radiotherapy, or ICIs: Zinc pyrophosphate nanoparticles ZnP@pyro; Mesoporous silica nanoparticles bMSNs loaded with neoantigen peptides, CpG, and photosensitizers; Nanoparticles carrying ICG/oxaliplatin; Metal-organic framework IDoi@TBC carrying IDO inhibitor; Core-shell nanoparticles PLGA-R837@Cat loaded with Cat and TLR7 agonist; Polymer nanovesicles Gem/Sul-NP loaded with gemcitabine and peptidyl-prolyl isomerase.	(178–190)
Simultaneously regulate NK cells and T cells	Promote the recruitment and activation of NK cells, and increase the infiltration and activity of CD8^+^ T cells	Zinc-sulfur@bovine serum albumin nanoclusters; Lipid nanoparticles delivering 5T4 and CD70 mRNA; Nanoparticles composed of aurora kinase inhibitor alisertib and JAK2 inhibitor ruxolitinib; RNA nanoparticles C-RNA-NPs.	(191–194)
Simultaneously regulate TAMs and T cells	Reduce M2 TAMs, reprogram TAMs phenotype, increase CD4^+^ T cells and CD8^+^ T cells, reduce Tregs	DNA nanodevices E64-DNA targeting TAMs lysosomes; Multivalent bispecific nanobioconjugates mBiNE; Nanovaccine stars NV; Nanoparticles based on polysaccharides (chitosan or hyaluronic acid): Nanogel D@HPMNG formed by coating with HA; Copper nanoparticles CuNP@PST based on galactomannan (PST001); CaP-PEG-HA@Ni/RSL3 formed by amorphous calcium phosphate modified with hyaluronic acid.	(195–198) (200)
Simultaneously regulate DCs and T cells	Promote the maturation of DCs, promote infiltration of CD4^+^ T cells and CD8^+^ T cells, activate CD8^+^ T cells, and inhibit Tregs	Nanoparticles composed of polysaccharides or other materials: Chitosan nanoparticles loaded with IR780 and IMQ; Multivalent mRNA vaccine formed by encapsulating mRNA encoding tumor neoantigens into mannose-modified chitosan ethyl choline bodies; Hydrogel formed by multifunctional sodium alginate; Dual drug delivery system LipEPAP18-APBA- HA loaded with epacadostat; Multifunctional nanoparticles HA/AIPH@ZIF-8 (HAZ) containing hyaluronic acid; Nanoparticles loaded with cisplatin and STING agonist SR-717; Micelles PPH@CR; Proteolysis-targeting chimera (PROTAC) formulation ARV@PEG-ICG.	(201–209)
Nanoparticles that affect various immune cells	Increase NK cells, promote DCs maturation, promote macrophage M1 polarization, promote infiltration of CD4^+^ T cells and CD8^+^ T cells, reduce MDSCs and Tregs	Nanoparticles composed of polysaccharides, gels, polymers, and many other materials: Chitosan oligosaccharide-dendritic polycarbonate nanoparticles CD NPs; Light-controlled polysaccharide nano immunomodulator Dex-NB-CPT; Sodium alginate hydrogel S1P-αPDL1@Ge; Thermosensitive hydrogel loaded with TGF-β inhibitor; Thermosensitive hydrogel loaded with disulfiram derivatives and Cu²^+^ (PLHCu); Multifunctional nano-epigenetic inhibitor CREDIT; PTEN mRNA nanoparticles; Targeted nanoparticles co-delivering IL-12 gene and CSF-1R inhibitor; Nanoparticle vaccine R848-GA@TCLs; Lipid calcium phosphate nanoparticles ppp-dsRNA; Liposome nanoparticles LNP-CDN loaded with cyclic dinucleotides; Virus-like particle nanovaccine N-M2T-gp100 HBc NV; Si9GM nanovaccine delivering αCLEC9A- antigen conjugates and STING agonists, etc.	(210–224)

## 9 Mechanisms of nanomedicine regulating immune cells

The physicochemical properties of drug-loaded nanomaterials along with the drugs they carry collectively determine their regulation of immune cells, the mechanisms may involve indirect regulation of immune cells (such as inducing tumor cells to undergo ICD, ferroptosis, or pyroptosis, and targeting tumor-derived exosomes) and direct regulation of immune cells (through activation of the cGAS-STING, NF-Kb, and mTOR pathways, as well as inhibition of SHP2, CD73, or glutaminase). In the following content, we will introduce the mechanisms by which nano-delivery systems regulate immune cells.

### 9.1 Indirectly regulate immune cells by affecting tumor cells

Most immunomodulatory nanoparticles primarily act on tumor cells to indirectly activate anti-tumor immunity, such as by inducing tumor cell death, including ICD, ferroptosis, and pyroptosis. For example, a multifunctional biomimetic nanoparticle platform Fe_3_O_4_@PDA@CaCO_3_-ICG@CM, consisting of CaCO_3_-modified magnetic polydopamine loaded with indocyanine green and wrapped with membranes from mouse lymphoma cells (EL4) expressing functional proteins (LFA-1; TGF-βR; PD-1; and FasL), can induce apoptosis of tumor cells, triggering ICD that promotes DCs maturation thereby activating CD4^+^/CD8^+^ T cells to eradicate primary tumors and inhibit distal tumors (225). Fe-doped and doxorubicin loaded HA@Cu_2-X_S-PEG (PHCN) nanomaterials were designed as targeted Fe-PHCN@DOX nano-reactors to maximize the effect of ICD to promote antigen presentation thereby stimulating T cell activation, in addition, Fe-PHCN@DOX can reprogram M2 TAMs into M1 TAMs by alleviating tumor hypoxia, at the same time T cell activation is promoted through blockade of the PD-1/PD-L1 immunosuppressive pathway by anti-PD-L1 nanoantibodies thereby achieving effective antitumor effects (226). In addition, the iron-containing metal-organic framework TPL@TFBF is formed through the coordination of tannic acid (TA) with Fe³^+^, loaded with triptolide (TPL) and coated with FA-modified BSA, among them TPL increases intracellular ROS production by inhibiting the expression of Nrf2, leading to ferroptosis and pyroptosis, releasing a large amount of DAMPs, which in turn stimulates DCs antigen presentation and CD4^+^/CD8^+^ T cell proliferation thereby suppressing tumor growth and metastasis (227). Moreover, I-124 labeled cancer cell membrane-mimicking nanovesicles 124I-P/C@CMLvs loaded with multi-flavonoid VI (PPVI) and CDDP, among them the loaded PPVI promotes p53 deubiquitination and stimulates ROS production thereby triggering ferroptosis amplification mediated by GPX4 signaling and pyroptosis mediated by the NLRP3/GSDMD/Caspase-1 axis, which significantly enhances the infiltration of immune cells in tumor tissues, including DCs, CD8^+^ T cells and CD4^+^ T cells, and further promotes tumor regression when combined with anti-PD-L1 therapy (228). In addition to indirectly regulating immune cells by inducing various forms of tumor cell death, targeting tumor-derived exosomes can also regulate immune cells. For example, engineered Escherichia coli Nissle 1917 (EcN) co-expressing anti-PD-L1 and anti-CD9 nanobodies (EcN-Nb) and modified with ICG-loaded zinc-based MOFs to generate EcN-Nb-ZIF-8CHO-ICG (ENZC), can specifically release nanobodies in response to NIR radiation thereby targeting tumor-derived exosomes (TDEs) to alleviate immunosuppression, such as reshaping TAMs to the M1 state and activating more effective and cytotoxic T lymphocytes, ultimately inhibiting tumor proliferation and metastasis (229). In summary, various nanoparticles have been shown to regulate immune cells through mechanisms related to their action on tumor cells which in turn indirectly regulate immune cells.

### 9.2 Directly regulate immune cells through signaling pathways or other mechanisms

Various nanoparticles have been shown to regulate immune cells and thereby activate antitumor immunity by activating the cGAS-STING pathway. For example, ROS-responsive nanoparticles self-assembled from a hybrid platinum prodrug CPT-Pt (IV), ROS-sensitive polymer, and mPEG2k-DSPE can accumulate at tumor sites and release cisplatin and CPT, leading to DNA double-strand damage, ultimately activating the cGAS-STING pathway, inducing DCs maturation and increasing tumor infiltration of CD8^+^ T cells (230). Moreover, manganese dioxide nanosheets loaded with Zebularine and surface-modified with hyaluronic acid to construct the nanoparticle Zeb@MH-NS can promote the accumulation of dsDNA in the cytoplasm upon reaching the tumor site, enhance the activation of the cGAS-STING signaling pathway, increase the production of type I interferons and the secretion of pro-inflammatory cytokines, and boost the maturation of DCs, the infiltration of CTLs, and the recruitment of NKs at the tumor site (231). In addition, DTSS NPs are formed by co-assembling salinomycin with thymopentin (TP5) and the ER-targeting photosensitizer s-780 and modified with DSPE-PEG-biotin, and the high heat and ROS generated by s-780 further induce ER stress thereby downregulating PD-L1 expression and activating the cGAS-STING pathway, while the loaded TP5 significantly promotes the proliferation and differentiation of T cells thereby enhancing anti-tumor immunity and inhibiting cancer metastasis and recurrence (232). Besides, doxorubicin-loaded manganese dioxide nanoparticles (MnO_2_) encapsulated in vitamin D_3_-inserted lipid-mixed neutrophil membranes to form the nanoplatform NED@MnO_2_-DOX can activate and enhance immune responses associated with the cGAS-STING pathway, such as promoting the secretion of type I interferon-β and pro-inflammatory cytokines, facilitating DCs maturation and promoting infiltration of CD8^+^ T cells into tumor tissues (233). In addition, the nanoscale activator based on Mn-quercetin coordination synthesized by mixing manganese tetrachloride tetrahydrate (MnCl_2_·4H_2_O) with an ethanol solution of quercetin, releases manganese ions that promote the generation of ROS, and enhances photothermal-induced DNA damage when combined with quercetin-induced apoptosis and further promotes the release of cytoplasmic DNA thereby activating the cGAS-STING pathway, enhancing immune activation and effectively inhibiting tumor growth (234). Furthermore, the cell membrane-coated ZIF-8@MnOx nano-platform mPDZM simultaneously loaded with doxorubicin and piperlongumine can effectively promote CD8^+^ T cell and NK cells infiltration by regulating SASP release, inducing ICD and activating the STING signaling pathway, while reducing Tregs and M2-like macrophages and can further enhance anti-tumor immunity and produce a strong abscopal effect on distant tumors when combinated with anti-PD-L1 therapy (235). Not only that, mesoporous organosilica nanoparticles loaded with omeprazole and phagocytosis inhibitors and surface-modified with hyaluronic acid can also promote ICD and activate the cGAS-STING pathway, leading to an increase in mature DCs and CD8^+^ T lymphocytes (236). In conclusion, various nanoparticles have been demonstrated to regulate immune cells in the tumor microenvironment and activate anti-tumor immunity by activating the cGAS-STING pathway.

In addition to the cGAS-STING pathway, nanoparticles can also regulate immune cells by activating other pathways and mechanisms. For example, the nanoparticle drug PAP-SeNPs constructed from SeNPs and red mushroom polysaccharide component (PAP-1a) can significantly activate the Tlr4/Myd88/NF-κB pathway, polarizing M2 macrophages into M1 macrophages thereby activating anti-tumor immune responses (237). In addition, the previously described CNPs-processed activated antigen-specific (P14) and non-specific CD8^+^ T cells can also produce more cytokines and release more effector molecules by activating the NF-κB signaling pathway, thereby enhancing the cytotoxic activity of CTLs (155). Not only that, the integrated probiotic expressing IL-15 (Bac) and pH-sensitive lysozyme nanoparticles (LYC) LYC@Bac can activate the mTOR pathway in NK cells, upregulate NKG2D to activate NK cells, and enhance granzyme and perforin secretion to strengthen cancer immunotherapy (238). In addition to regulating immune cells by activating pathways, nanoparticles can also participate in the regulation of immune cells through various mechanisms. For example, nano-PROTACs composed of a photosensitizer (protoporphyrin IX, PpIX) and a Src homology 2 domain-containing phosphatase 2 (SHP2)-targeting PROTAC peptide (aPRO) connected by a caspase-3 cleavable linker, among them aPRO is activated after phototherapy due to the increased expression of caspase-3 in tumor cells, inducing the targeted degradation of SHP2 through the ubiquitin-proteasome system, and the persistent depletion of SHP2 blocks the immunosuppressive checkpoint signaling pathways CD47/SIRPα and PD-1/PD-L1 thereby reactivating anti-tumor macrophages and T cells (239). Metabolic immune checkpoint ecto-5’-nucleotidase (CD73) significantly inhibits the function of anti-tumor T cells and increases the activity of immunosuppressive cells by regulating the imbalance of extracellular adenosine (ADO), while multifunctional Au@Cu_2-x_Se nanoparticles (ACS NPs) significantly inhibit the expression of CD73 thereby reducing ADO production, lowering ADO-driven immunosuppression and enhancing the infiltration and activity of anti-tumor T cells in tumors (240). In addition, a purpurin-copper coordinated nanoplatform TPGS/PC@Ce6 NPs, in which purpurin reprograms glutamine metabolism via glutaminase inhibition, promotes DCs maturation, and enhances tumor immunotherapy (241). In summary, nanoparticles can also directly regulate immune cells in the tumor microenvironment by activating the NF-κB or mTOR pathways, as well as by inhibiting SHP2, CD73, or glutaminase.

## 10 Discussion

Cancer is the leading cause of death and immunotherapy has attracted a wide range of attention due to its advantages such as durable response, long-term survival benefit, and lower toxicity and side effects (242, 243), in which immune cells are the target cells for immunotherapy, which as part of the TME are mainly composed of TAMs, DCs, NK cells, TANs, MDSCs and T cells. With the progression of tumors, the number of anti-tumor immune cells such as DCs, NK cells, M1 TAMs, and N1 TANs in TME decreases and their functions are suppressed while the number of immunosuppressed cells M2 TAMs, MDSCs, N2 TANs, and eTregs increases, resulting in tumor cells evading surveillance by the immune system and growing indefinitely. A large number of studies have shown that immune cells play a vital role in the initiation, development, recurrence and anti-tumor therapy of tumors, so the treatment of immune cells that are not considered is not sufficient in the treatment of tumors, and the strategy of targeting immune cells to treat tumors can be achieved by increasing anti-tumor immune cells or their functions, reducing immunosuppressive cells or weakening their functions.

With the extensive development and application of drug-loaded nanosystems, a large number of studies have confirmed that drug-loaded nanoparticles have the effect of synergizing and reducing toxicity in the treatment of tumors, and nanoparticle drug delivery systems have become an effective solution to various obstacles in the delivery of anti-cancer drugs, anti-cancer natural products, and anti-cancer genes (244). And a large number of studies have confirmed that nanoparticles can restore anti-tumor immunity by modulating immune cells in the TME, such as regulate TAMs (including deplete M2 TAMs in the TME, limit monocyte recruitment and localization to tumor tissues, and program M2 TAMs to M1 TAMs), NK cells (including enhancing NK cells homing or enhancing NK cells function), DCs (including increasing the number of DCs or promoting their maturation), TANs (including reducing neutrophils at tumor sites, enhancing the antitumor activity of neutrophils, as well as altering neutrophil polarity), MDSCs (including reducing circulating and tumor-infiltrating MDSCs, altering MDSC phenotype or inhibiting MDSC function, caused by nanoparticles composed of nanocapsules and metal-organic frameworks, micelles and polymers, dendrimers, liposomes, and other materials), T cells (including increasing CD4^+^ T or CD8^+^ T cells infiltration or their activity, or reducing the number of Tregs, caused by liposomes, gels, cerium and selenium nanoparticles, hyaluronic acid-modified nanoparticles, nanocapsules, nanovesicles, nanoparticles of other materials, as well as the combination of nanoparticles with other therapies) and simultaneously regulating multiple immune cells. However, the TME is complex, including not only various immune cells but also cellular and non-cellular components such as mesenchymal cells, blood vessels, lymphatic vessels, and cytokines (245), therefore, it is also possible that nanoparticles that exert anti-tumor effects by modulating other components.

In addition, there are still many problems with nanoparticle drug therapies targeting immune cells for tumors, including but not limited to the following: (1) A large number of products are difficult to reproduce due to the synthesis and post-modification procedures of nanoparticles, especially highly engineered nanoparticles, and this also result in increased costs (246). (2) Nanoparticles have more complex formulations compared with small-molecule drugs, and their physicochemical properties, such as size, structure, composition, and surface characteristics, all affect the form of nanomedicine preparations in the body, moreover, the physicochemical characteristics of nanoparticles change significantly after administration due to interactions with biomolecules (such as protein coronas), making the evaluation of their *in vivo* performance difficult (247). (3) Nanoparticles or their degradation fragments may lead to harmful nano-bio interactions thereby inducing nanotoxicity, including genotoxicity, immunotoxicity, neurotoxicity, pulmonary toxicity and vascular dysfunction (248). Among these, the *in vivo* immunotoxicity caused by nanoparticles mainly includes blood immunotoxicity, tissue inflammation, and immune organ damage. Nanoparticles can also cause mitochondrial and lysosomal damage, endoplasmic reticulum stress and Golgi apparatus rupture. Moreover, nanoparticles within immune cells may trigger genotoxicity and epigenetic toxicity, and these immunotoxic effects are still observed six months to one year after administration although the particles and their degradation products may be cleared within two months (249). (4) Compared with spontaneously formed cancer tissues in humans, cancer tissues in animal models lack the ability to reproduce the characteristics of human malignant tumors, which may lead to differences between the therapeutic effects observed in preclinical studies and clinical efficacy. It is also necessary to study their physicochemical properties and potential degradation patterns *in vivo*, focusing on the distribution and clearance of nanoparticles and their long-term effects on various immune cells. (5) Although many studies show that a large number of nanoparticles can exert anti-tumor effects by modulating immune cells in the tumor microenvironment, there is no comparison between nanomaterials to indicate which material’s nanoparticles are more advantageous. (6) Currently, the global regulatory policies for nanomedicines are still imperfect which may affect the process of translating compliant nanomedicines into clinical applications. In summary, many drug-loaded nanosystems still face numerous issues in clinical translation, however, based on the synergistic and detoxifying advantages of nanoparticles in tumor therapy and the important role of immune cells in tumor occurrence and development, along with the continuous improvement of drug-loaded nanosystems and the refinement of relevant policies, nanomedicine delivery systems are bound to become a direction in tumor treatment. Moreover, nanoparticle therapies targeting immune cells will become an important strategy for tumor therapy, with broad application prospects in the field of tumor treatment.

## Glossary

TME: Tumor microenvironment
TAMs: Tumor-associated macrophage
NK: Natural killer
DC: Dendritic cell
TANs: Tumor-associated neutrophils
MDSCs: Myeloid-derived suppressor cells
MMP-2: Matrix metalloprotein-2
ICD: Immunogenic death
CSF-1R: Colony-stimulating factor 1 receptor
siRNA: Small interfering RNA
PEG: Polyethylene glycol
CCL2: CC motif chemokine 2
VEGF: Vascular endothelial growth factor
CCR2: CC motif chemokine receptor-2
TLR: Toll-like receptors
TAMM: Tumor-associated macrophage membranes
TGF-β: Transforming growth factor β
IFN-γ: Interferon-γ
SeNP: Selenium nanoparticles
NKG2D: Natural killer cell group 2D
shRNA: Short hairpin RNA
CTLs: Cytotoxic T lymphocytes
Ce6: Photosensitizer chlorine E6
NIR: Near-infrared
MOF: Metal-organic framework
G-MDSC: Granulocyte myeloid-derived suppressor cells
PI3Kγ: Phosphatidylinositol 3-kinase γ
ICD: Immunogenic cell death
ROS: Reactive oxygen species
CXCR4: C-X-C motif chemokine receptor-4
M-MDSC: Monocyte myeloid-derived suppressor cells
TAD: Tadalafil
ICG: Indole green
APC: Antigen-presenting cells
ACT: Adoptive T cell transfer
LNP: Liposomal nanoparticles
GM-CSF: Granulocyte-macrophage colony-stimulating factor
G-CSF: Granulocyte colony-stimulating factor
CTLA-4: Cytotoxic T lymphocyte-associated protein 4
IDO1: Indoleamine 2,3-dioxygenase-1
DOX: Doxorubicin
PTT: Photothermal therapy
PDT: Photodynamic therapy
ICI: Immune checkpoint inhibitors
PD-1: Programmed cell death 1
PD-L1: Programmed death-ligand 1
MM: Multiple myeloma
HER2: Human epidermal growth factor receptor 2
PTEN: Phosphate and tension homology deleted on chromsome ten
MPE: Malignant pleural effusion
IL: Interleukin
R848: Resiquimod
